# Improved health by combining dietary restriction and promoting muscle growth in DNA repair‐deficient progeroid mice

**DOI:** 10.1002/jcsm.13570

**Published:** 2024-09-08

**Authors:** Wilbert P. Vermeij, Khalid Alyodawi, Ivar van Galen, Jennie L. von der Heide, María B. Birkisdóttir, Lisanne J. van't Sant, Rutger A. Ozinga, Daphne S.J. Komninos, Kimberly Smit, Yvonne M.A. Rijksen, Renata M.C. Brandt, Sander Barnhoorn, Dick Jaarsma, Sathivel Vaiyapuri, Olli Ritvos, Tobias B. Huber, Oliver Kretz, Ketan Patel

**Affiliations:** ^1^ Princess Máxima Center for Pediatric Oncology Utrecht Netherlands; ^2^ Oncode Institute Utrecht Netherlands; ^3^ School of Biological Sciences University of Reading Reading UK; ^4^ College of Medicine Wasit University Kut Iraq; ^5^ III. Department of Medicine University Medical Center Hamburg‐Eppendorf Hamburg Germany; ^6^ Hamburg Center for Kidney Health (HCKH) Hamburg Germany; ^7^ Department of Neuroscience Erasmus University Medical Center Rotterdam Rotterdam Netherlands; ^8^ Department of Molecular Genetics, Erasmus MC Cancer Institute Erasmus University Medical Center Rotterdam Rotterdam Netherlands; ^9^ School of Pharmacy University of Reading Reading UK; ^10^ Department of Physiology University of Helsinki Helsinki Finland

**Keywords:** Ageing, Dietary restriction, Kidney, Muscle, Myostatin, Progeria

## Abstract

**Background:**

Ageing is a complex multifactorial process, impacting all organs and tissues, with DNA damage accumulation serving as a common underlying cause. To decelerate ageing, various strategies have been applied to model organisms and evaluated for health and lifespan benefits. Dietary restriction (DR, also known as caloric restriction) is a well‐established long‐term intervention recognized for its universal anti‐ageing effects. DR temporarily suppresses growth, and when applied to progeroid DNA repair‐deficient mice doubles lifespan with systemic health benefits. Counterintuitively, attenuation of myostatin/activin signalling by soluble activin receptor (sActRIIB), boosts the growth of muscle and, in these animals, prevents muscle wasting, improves kidney functioning, and compresses morbidity.

**Methods:**

Here, we investigated a combined approach, applying an anabolic regime (sActRIIB) at the same time as DR to *Ercc1* ^Δ/−^ progeroid mice. Following both single treatments and combined, we monitored global effects on body weight, lifespan and behaviour, and local effects on muscle and tissue weight, muscle morphology and function, and ultrastructural and transcriptomic changes in muscle and kidney.

**Results:**

Lifespan was mostly influenced by DR (extended from approximately 20 to 40 weeks; *P* < 0.001), with sActRIIB clearly increasing muscle mass (35–65%) and tetanic force (*P* < 0.001). The combined regime yielded a stable uniform body weight, but increased compared with DR alone, synergistically improved motor coordination and further delayed the onset and development of balance problems. sActRIIB significantly increased muscle fibre size (*P* < 0.05) in mice subjected to DR and lowered all signs of muscle damage. *Ercc1* ^Δ/−^ mice showed abnormal neuromuscular junctions. Single interventions by sActRIIB treatment or DR only partially rescued this phenotype, while in the double intervention group, the regularly shaped junctional foldings were maintained. In kidney of *Ercc1* ^Δ/−^ mice, we observed a mild but significant foot process effacement, which was restored by either intervention. Transcriptome analysis also pointed towards reduced levels of DNA damage in muscle and kidney by DR, but not sActRIIB, while these levels retained lower in the double intervention.

**Conclusions:**

In muscle, we found synergistic effects of combining sActRIIB with DR, but not in kidney, with an overall better health in the double intervention group. Crucially, the benefits of each single intervention are not lost when administered in combination, but rather strengthened, even when sActRIIB was applied late in life, opening opportunities for translation to human.

## Introduction

The global rise in human life expectancy, besides extending healthy lifespan, has led to an increased prevalence of age‐associated co‐morbidities, including diabetes, cachexia, sarcopenia, chronic kidney disease, and many neurodegenerative diseases. Ageing is a complex multifactorial process, with numerous detrimental effects occurring at the cellular (accumulation of damaged macromolecules and intracellular debris), organ (e.g., impaired tissue renewal), and whole organismal level (changes in hormonal, serological, and inflammatory status).^1^, ^2^ All hallmarks of ageing (e.g., cellular senescence, loss of proteotoxicity, mitochondrial dysfunction, and stem cell exhaustion) could, in some degree, follow from DNA damage.^3^, ^4^ The role of damage accumulation in ageing and age‐associated co‐morbidities especially became apparent in two major patient cohorts. Firstly, cancer patients treated with radio/chemotherapy treatment while saving lives nevertheless induces DNA damage that later results in various late‐life effects and features of accelerated ageing.^5^ Secondly, progeroid syndromes characterized by segmental and rapid ageing, for example, Cockayne syndrome (CS) and trichothiodystrophy (TTD), are caused by mutations in genes controlling nuclear integrity and DNA repair.^6^ Importantly, mutant mouse models carrying genetic defects of these progeroid syndromes reproduce many symptoms found in patients and model many aspects of normal ageing.

*Ercc1* ^Δ/−^ mice that are deficient for the DNA repair nuclease cofactor ERCC1 (excision repair cross complementing‐group 1) have been shown to represent a particularly powerful accelerated ageing model.^7^, ^8^ *Ercc1* ^Δ/−^ mice lack one functional *Ercc1* allele while the other allele encodes a seven amino acid C‐terminally truncated ERCC1 variant impairing the interaction with XPF, resulting in strongly reduced residual ERCC1/XPF activity and increased sensitivity to DNA damage.^7^, ^9^ Consequently, a broad spectrum of DNA lesions accumulates more rapidly, causing severe progressive wide‐spread ageing, mimicking aspects of segmental ageing from various human progeroid conditions including CS, TTD, and Fanconi anemia.^8^ The lifespan of *Ercc1* ^Δ/−^ mice is significantly shortened to 4–6 months,^10^, ^11^ and within that time span, they show numerous progressive premature ageing phenotypes, including decreased body weight, prominent global neurodegeneration (dementia, ataxia, and neuronal plasticity), osteoporosis, cardiovascular, immunological ageing, thymic involution, cachexia, sarcopenia, early infertility, liver and kidney ageing, and so on.^8^ These changes are accompanied by progressive behavioural‐physiological‐hormonal alterations, increased cellular senescence, overall frailty, and gene expression patterns like natural ageing. *Ercc1* ^Δ/−^ mice have been fundamental for the identification of tissues affected in the human progeroid DNA repair‐deficiency syndromes, but also for unravelling mechanisms of natural ageing.^3^, ^7^, ^8^, ^12^, ^13^, ^14^

To counteract ageing and age‐related conditions, for example, cachexia and sarcopenia, various strategies have been explored. Most interventions that modulate health/lifespan seem to converge on the balance between nutritional intake and exercise, or related metabolic and signalling processes.^1^ Dietary restriction (DR) is a frequently studied long‐term nutritional intervention in which food intake is generally reduced by approximately 30%. It extends lifespan in numerous species (including rodents and non‐human primates), retards many symptoms of ageing, and overall provides broader and more pronounced benefits compared with DR mimetics.^1^, ^10^, ^15^, ^16^, ^17^ DR lowers body weight and affects numerous intracellular and intercellular processes, which include nutrient‐ and energy‐sensing pathways and boosts overall resilience capacity of an organism.^1^, ^18^, ^19^ While DR extends lifespan by 30% in wildtype rodents, it more than doubles median and maximal lifespan of *Ercc1* ^Δ/−^ mice.^12^, ^20^ Furthermore, 30% DR strongly delayed accelerated ageing in multiple organ systems, most notable in neurological functioning,^12^, ^21^ although the effect on skeletal muscle was yet not investigated.

An intervention specifically preventing skeletal muscle atrophy is based on inhibiting activin/myostatin‐signalling. sActRIIB, a ligand trap capable of sequestering activin and myostatin, was found to increase total body weight of *Ercc1* ^Δ/−^ mice due in large part to an increase in muscle mass.^22^ Not only were muscles heavier but also showed improved contractile properties following attenuation of activin/myostatin‐signalling. Profoundly, sActRIIB treatment compressed morbidity to almost the last few days of life in *Ercc1* ^Δ/−^ mice but had no impact on lifespan. In addition, the sActRIIB‐injection also prevented premature ageing of the kidneys, liver, and skeletal system.^22^ Whether this was a direct effect of sActRIIB or mediated via interorgan crosstalk from the skeletal muscles is under investigation.

Hence, there are two robust means of altering the (skeletal muscle) ageing process of *Ercc1* ^Δ/−^ mice. One, DR suppresses organismal growth and extends lifespan but has no impact on altering the age‐related catabolic pathways, while the other dampens activin/myostatin‐signalling to boost muscle growth and promote health‐span in an anabolic response without affecting longevity.

We hypothesize benefits at the organismal level imbued by DR can be further enhanced by sActRIIB treatment especially through the latter's ability to promote muscle structure and function. We present for the first‐time beneficial evidence for combining the two treatments at both the tissue and physiological level.

## Methods

### Mouse model and interventions

The generation and characterization of *Ercc1* ^∆/−^ mice has been previously described.^9^, ^12^ Briefly, *Ercc1* ^∆/+^ animals (in pure C57BL6J or FVB backgrounds) were crossed with *Ercc1* ^+/−^ mice (in opposite genetic‐backgrounds) to yield *Ercc1* ^∆/−^ offspring with a genetically uniform F1‐hybrid C57BL6J/FVB background. Wild‐type F1‐littermates were used as controls.

Experiments were performed in accordance with the Principles of Laboratory Animal Care and the guidelines approved by the Dutch Ethical Committee in full accordance with European legislation (permit #139‐12‐13, 139‐12‐18, and 18‐6886‐05).

All animals were randomly divided over the intervention groups and housed in individual ventilated cages under specific‐pathogen‐free‐conditions with a maintained environment (20–22°C with 12 h‐light: 12 h‐dark‐cycles). Mice were visually inspected daily and were weekly weighed and scored blindly for gross morphological and motor abnormalities. Animals were bred and maintained on AIN93G synthetic pellets (Research‐Diet‐Services, Netherlands).

DR was applied as 30% food reduction as previously published.^12^ Mice on average ate 2.3 g/day, resulting in 1.6 g/day for 30% DR, which was kept constant throughout the experiment. Water was freely available. Myostatin/activin‐block was applied through intraperitoneal injections with 10 mg/kg of sActRIIB‐Fc twice every week as previously published.^22^

### Muscle histology and tension measurements

Muscles were dissected, weighted, and frozen onto liquid nitrogen‐cooled isopentane; 10 μm cryosections were processed for DHE and SDH staining and immunohistochemistry as previously published^22^ as well information in the supporting information and with antibodies given in Table‐S1.

EDL muscles in oxygenated Kreb's solution were tied to 1200A Muscle Test System (Aurora‐Scientific, Ireland), and contractions were induced by applying voltage for 500 ms at 20, 50, 100, and 200 Hz as previously published.^22^

### Transmission electron microscopy

For electron microscopy small pieces of mouse renal cortex and biceps muscle (*n* = 3/group) were immersion fixed in 4% PFA in 0.1 M PB. Tissue blocks were stained with OsO_4_ and uranyl acetate, dehydrated and embedded in epoxy resin. Ultrathin sections were analysed using a Philipps CM100 transmission electron microscope and Olympus ITEM software.

### RNA‐seq

RNA from quadriceps muscle and kidneys from *Ercc1* ^Δ/−^ and WT animals treated with DR and/or sActRIIB was used for transcriptome analysis according to previous procedures.^23^ Analysis of raw data files was performed on our in house‐generated data analysis pipeline.^23^ An extended description is provided as supporting information. All data files have been submitted to the NCBI gene expression omnibus under GSE268971.

### Statistical analysis

Statistical analyses were performed using GraphPad Prism (San Diego, CA, version 10.1.0). One‐way ANOVA with Tukey's multiple comparison was used for analyses between genotype and interventions with multiple groups except muscle fibre area and profile for which we used two‐way ANOVA. Area under the curve for bodyweight and food intake was calculated as log10‐values between indicated weeks. Log‐rank (Mantel–Cox) tests were used to compare curves for onset of tremors, imbalance and survival. Hazard plot shows log values of hazard ratio (log‐rank) compared with controls and 95% confidence interval. Graphs illustrate individual values, means and standard error unless otherwise indicated. *P < 0.05, **P < 0.01, ***P < 0.001, ****P < 0.0001.

## Results

### Ercc1^Δ/−^ lifespan with combined dietary restriction and activin ligand trap

**Figure 1:**
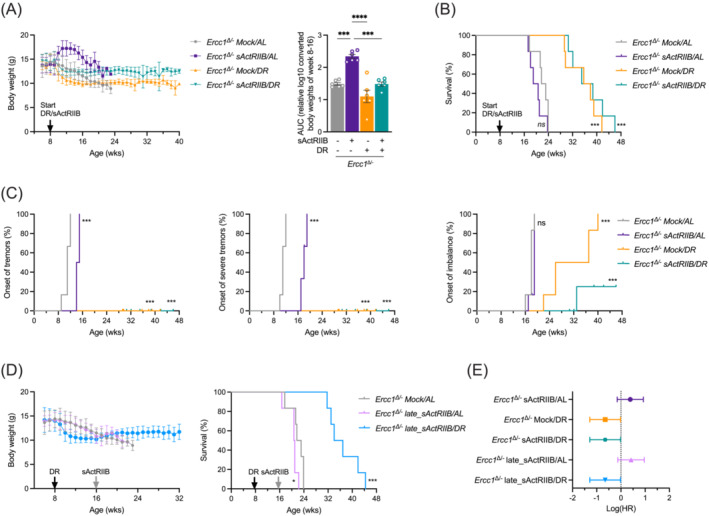
Whole body assessment of sActRIIB, dietary restriction and dual intervention on *Ercc1* ^Δ/−^ mice. (A) Mean body weights development (±SD) and area under the curve (AUC) of body weight (as mean ± SE and individual data points) of *Ercc1* ^Δ/−^ mice under sActRIIB and/or dietary restriction (DR) conditions versus mock‐treated ad libitum (AL) fed. All treatments were initiated from 8 weeks of age (black arrow). (B) Survival curves of *Ercc1* ^Δ/−^ mice across the different intervention groups. (C) Onset of neurological abnormalities tremors, severe tremors, and imbalance with age. (D) Body weight changes and lifespan curves of *Ercc1* ^Δ/−^ mice under AL and DR conditions when sActRIIB was administered late in life from 16 weeks of age (grey arrow). (E) Forest plot of effect size for the logarithm of the hazard ratio (HR; with 95% confidence interval as error bars) for changes in survival of all cohorts. *n* = 6 animals per group. *P < 0.05, ***P < 0.001, ****P < 0.0001.

To examine the combinatorial effects of sActRIIB and dietary restriction (DR) in *Ercc1* ^Δ/−^ mice, we first performed a lifespan analysis with four groups; both interventions alone, combined, and control untreated. We subjected *Ercc1* ^Δ/−^ mice to sActRIIB or mock treatment via I.P. injections (two‐times weekly) from 8 weeks of age, a time when limited signs of ageing are observed in these animals. Simultaneously, animals had *ad libitum* (AL) access to food or were subjected to a 30% DR regimen, based on their average food intake measured before.^12^ In line with previous reports,^12^, ^22^ we found that injection of sActRIIB caused a significant increase in body weight in both male and female *Ercc1* ^Δ/−^ mice, while DR induced a lower but uniformly stable body weight. Notably, sActRIIB also increased weight of DR *Ercc1* ^Δ/−^ mice (Figures 1A and S1A–D). As previously reported, DR dramatically extended lifespan in *Ercc1* ^Δ/−^ mice. Supplementation with sActRIIB to the DR regime did not shorten lifespan (Figures 1B and S1E,F).

Longitudinal examination of phenotypical readouts showed that sActRIIB treatment in AL‐fed *Ercc1* ^Δ/−^ mice delayed the onset of tremors and reduced tremor severity but had no effect on the onset of imbalance (Figure 1C). Instead, the onset of imbalance was greatly postponed by sActRIIB in DR‐treated *Ercc1* ^Δ/−^ mice (*P* = 0.024) beyond the level of delay already triggered by DR alone, while signs of tremors were absent in all DR *Ercc1* ^Δ/−^ mice (Figure 1C).

To assess the possibility of treating muscle wasting late in life, we repeated our lifespan study of combining DR and activin ligand trap, but this time administered sActRIIB from 16 weeks of age, when the animals severely lost body weight and clear signs of ageing are present. Although signs of behavioural abnormalities were unchanged by late‐life sActRIIB treatment, body weight was still increased in both AL‐fed and DR‐treated animals (Figures 1D and S1G–I). Yet no significant impact on lifespan was noted beyond the effects observed by DR (Figure 1D,E).

These data show that sActRIIB treatment, when initiated before the onset of ageing features, retards phenotypical deficits in both AL and DR *Ercc1* ^Δ/−^ mice. Furthermore, it increases body weight without impacting negatively on benefits of DR when initiated after the onset of ageing phenotypes.

### Behaviour, muscle, and organ weight changes induced by the combined sActRIIB + dietary restriction treatment

**Figure 2:**
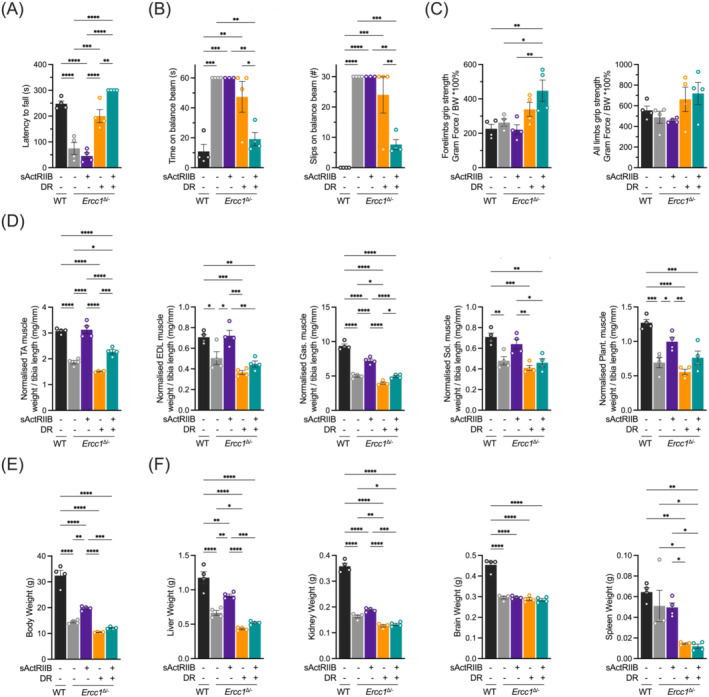
Exercise profiling of *Ercc1* ^Δ/−^ mice and organ weights following sActRIIB, dietary restriction and combined at 16 weeks of age. (A) Average time spent on an accelerating rotarod of wild‐type and *Ercc1* ^Δ/−^ mice on different interventions at 16 weeks of age. (B) Motor coordination on the balance beam indicated by time to cross the beam and the number of missteps made during. (C) Grip strength measure of forelimbs and all limbs normalized to body weight. (D) Dissected muscle weights for tibialis anterior (TA), extensor digitorum longus (EDL), gastrocnemius (Gas.), soleus (Sol.), and plantaris (Plant.) normalized to tibia length. (E) Body weight and (F) indicated organ weights at week 16. *n* = 4 animals per group. Mean ± SE and individual data points are indicated. *P < 0.05, **P < 0.01, ***P < 0.001, ****P < 0.0001.

To follow up on the synergistic benefits of imbalance onset, we started a second cohort of mice subjected to both sActRIIB and/or DR from 8 weeks of age and further assessed additional behavioural parameters at 16 weeks of age. Skilled locomotor function, measured as time spend on an accelerating rotarod, was significantly enhanced by DR and further improved by sActRIIB injection (Figure 2A). In addition, locomotion on a balance beam, both as time spend to cross and number of missteps during, was mostly improved by the combination treatment (Figure 2B). Muscle strength, as determined with a grip strength meter, was moderately increased by the double intervention (Figures 2C and S2A,B).

Next, we investigated the tissues responsible for body weight changes by first focusing on skeletal muscle, as previous work has shown that this tissue is highly responsive to both interventions.^16^, ^22^, ^24^, ^25^ Accordingly, we found that in tibialis anterior (TA), extensor digitorum longus (EDL), gastrocnemius (Gas.), soleus (Sol.), and plantaris (Plant.) muscle, the combination treatment led to an increase in muscle mass compared with DR alone (Figures 2D and S2C–L), an effect that was independent of metabolic and contractile properties in skeletal muscle. Whole body weights showed the same trend as for muscles (Figure‐2E). Investigations into the impact of the treatments on other organ weights and blood parameters revealed a picture that greatly differed when compared with their outcome on skeletal muscle (Figures 2F and S3). Herein, there was a general trend that organs from *Ercc1* ^Δ/−^ mice were lighter than those of WT. Secondly, sActRIIB treatment made little significant difference compared with untreated *Ercc1* ^Δ/−^ mice. Organ weights from DR *Ercc1* ^Δ/−^ mice were lighter than AL‐fed counterparts and fasting blood glucose levels lower. Lastly, introduction of sActRIIB to DR *Ercc1* ^Δ/−^ mice had no major impact compared with the DR treatment alone (Figures 2F and S3).

These data show that a DR regime results in a decreased mass of most organs and a general decline in overall body size. However, sActRIIB treatment when combined with a DR regime was able to induce muscle growth but had very little effect on other organs compared with DR alone.

### Profiling of muscle fibres following both single and combined treatments

**Figure 3:**
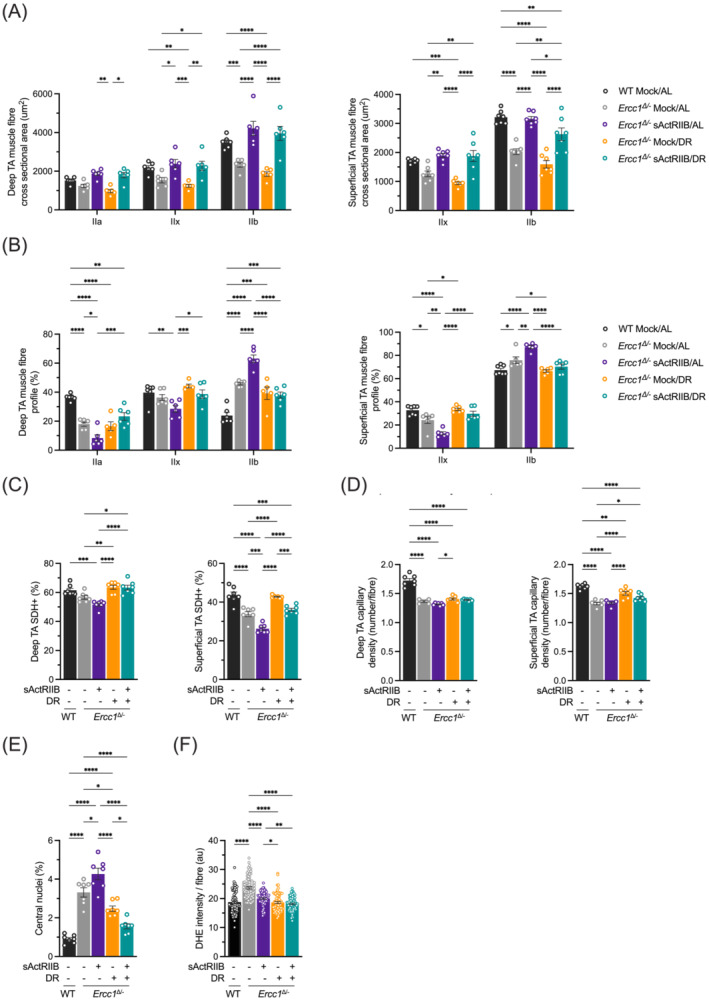
Profiling of *Ercc1* ^Δ/−^ muscle fibres following dual intervention. (A) MHCIIA/IIB fibre size profile of deep and superficial regions of TA muscle. (B) MHCIIA/IIB fibre frequency profile of deep and superficial regions of TA muscles. Between 60–80 fibres were counted from each mouse before being averaged per cohort. (C) SDH profiling of deep and superficial regions of TA muscles. (D) Capillary density profile, through CD31 staining, of deep and superficial regions of TA muscles. (E) Quantification of regenerating fibres in the EDL through counting of centrally located nuclei. (F) Measure of oxidate damage in EDL. All muscles are from mice at 16 weeks of age. *n* = 8 WT Mock/AL, *n* = 7/8 *Ercc1* ^Δ/−^ Mock/AL, *n* = 7/8, *Ercc1* ^Δ/−^ sActRIIB/AL, *n* = 7/8, *Ercc1* ^Δ/−^ Mock/DR, *n* = 7/8 *Ercc1* ^Δ/−^ sActRIIB/DR. *P < 0.05, **P < 0.01, ***P < 0.001, ****P < 0.0001.

Having detected an increase in muscle mass when comparing the double intervention to a DR alone regime, we focused on elucidating their impact on its structure and function. We found that irrespective of muscle group or portion of the muscle, the combined intervention led to a significant increase in muscle fibre size when compared with DR alone (Figure 3A, S4A). Thereafter, we focused on the TA for more detailed examination. Interestingly, the composition of the muscle was changed by the interventions. Generally, *Ercc1* ^Δ/−^ had fewer slow MHC fibres than WT which was further decreased by sActRIIB treatment (Figure 3B; for a mechanistic explanation, see Alyodawi et al.^22^). DR treatment maintained the elevated number of non‐IIB fibres compared with *Ercc1* ^Δ/−^ muscle. Interestingly, dual treatment maintained non‐IIB MHC fibres to levels of DR treatment alone (Figure 3B). Similar results were found in EDL‐(Figure S4B). In summary, dual treatment results in the formation of large slow fibres compared with the single DR intervention.

Next, we assessed metabolic consequences of the interventions by quantifying the expression of succinate dehydrogenase (SHD), an enzyme with high activity in fibres with high oxidative metabolism. Herein, we found that the deficit in oxidative metabolism induced by sActRIIB treatment was corrected by DR (Figure 3C). These changes were partially mirrored by capillary density in the TA muscle. Herein, there was a significant increase in capillary density in the *Ercc1* ^Δ/−^ DR group compared with sActRIIB, whereas the dual intervention was in‐between (Figure 3D).

Interestingly, the quantification of muscle damage indicated by fibres with centrally located nuclei found that the dual intervention was most effective in preventing these events. Indeed, the dual treatment has the number of regenerating fibres that were at levels close to those from WT mice (Figure 3E). Lastly, we quantified the level of oxidative damage by assessing the degree of DHE intensity. These experiments revealed that all three interventions were able to reduce damage by reactive oxygen species (Figure 3F).

In summary, combining sActRIIB with DR had a beneficial effect on muscle fibre growth while imbuing them with oxidative features and being resilient to damage.

### Preserved muscle function and ultrastructure by combined sActRIIB + dietary restriction treatment

**Figure 4:**
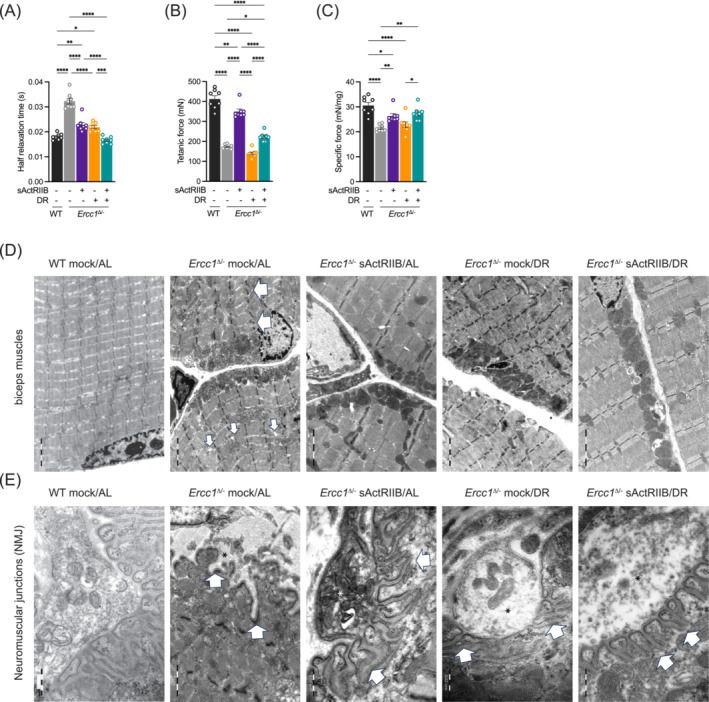
Impact of dual intervention on *Ercc1* ^Δ/−^ muscle function and ultrastructure. *Ex‐vivo* assessment of (A) half relaxation time and (B) tetanic and (C) specific force. All muscles are from mice at 16 weeks of age. *n* = 8 WT Mock/AL, *n* = 7/8 *Ercc1* ^Δ/−^ Mock/AL, *n* = 7/8, *Ercc1* ^Δ/−^ sActRIIB/AL, *n* = 7/8, *Ercc1* ^Δ/−^ Mock/DR, *n* = 7/8 *Ercc1* ^Δ/−^ sActRIIB/DR. (D) sActRIIB but not dietary restriction prevents *Ercc1* ^Δ/−^ muscle ultrastructural abnormalities. All transmission electron microscopy (TEM) images are from biceps muscle. In *Ercc1* ^Δ/−^ mice, biceps muscles display disorganized, split sarcomeres of high variable width frequently containing a disrupted M‐Line. Moreover, the sarcoplasmic reticulum of these muscles was dilated. sActRIIB‐treatment alone as well as in combination with dietary restriction rescues this phenotype completely. In contrast, dietary restriction alone has no effect on any ultrastructural changes observed in *Ercc1* ^Δ/−^ mice. Large arrows indicate disorganized sarcomeres and small arrows dilated sarcoplasmatic reticulum. (E) Neuromuscular junctions (NMJ) of biceps muscles in *Ercc1* ^Δ/−^ mice display and obvious phenotype at the postsynaptic side. Namely in *Ercc1* ^Δ/−^ mice the postsynaptic junctional folds of the basal lamina are almost completely absent or appear rudimentary. sActRIIB or dietary restriction could only partially rescue this phenotype, that is, the distance between the junctional foldings is still highly variable as well as their depth and width. Only double intervention by sActRIIB and dietary restriction fully restores the NMJ phenotype observed in *Ercc1* ^Δ/−^ mice. *P < 0.05, **P < 0.01, ***P < 0.001, ****P < 0.0001.

Having detected numerous signs that the muscle was impacted positively at the cellular and molecular level by the dual intervention, we examined its impact on function. Our data using the forelimb grip strength approach showed that the dual intervention resulted in highest degree of force generation. However, this approach impacts not only the ability of muscle to generate force but also volition. We therefore carried out ex vivo force field stimulation measures that report directly on contractile properties of muscle. Previously, we have shown that the contraction characteristics of muscle from *Ercc1* ^Δ/−^ mice were significantly changed exemplified by an increase in the time taken from muscle to relax after electrical stimulation.^22^ Again, these findings were reproduced in this study. Here, we found that DR alone reduced the relaxation time which was further improved by the dual DR + sActRIIB intervention (Figure 4A). Critically, both tetanic and specific force were higher in dual treatment compared with DR alone (Figure 4B,C).

The ultrastructure of skeletal muscle following the different interventions was examined using transmission electron microscopy. Numerous abnormalities were evident in the muscle from *Ercc1* ^Δ/−^ mice including heterogeneous Z‐line lengths, missing Z‐lines, misaligned Z‐lines, split sarcomeres, and dilated sarcoplasmic reticulum (Figure 4D). These abnormalities were largely absent in muscle from *Ercc1* ^Δ/−^ mice treated with sActRIIB alone or with sActRIIB in combination with DR. In contrast, DR alone did not rescue the ultrastructural phenotype of skeletal muscles in *Ercc1* ^Δ/−^ mice (Figure 4D).

In addition, we investigated the ultrastructure of the neuromuscular junction (NMJ), a structure known to be involved in muscle ageing and sarcopenia. Previously, immunofluorescence analysis showed that about 25% of the *Ercc1* ^Δ/−^ NMJs are abnormal at 16 weeks, either being partially innervated, poly‐innervated or fully denervated.^11^ Surprisingly and in contrast to previous findings, the sciatic nerve (data not shown) and the axon terminals (Figure 4E, asterisk) at the NMJ showed no obvious pathological phenotype. However, in NMJs of *Ercc1* ^Δ/−^ mice, the junctional folding of the basement membrane at the postsynaptic side was either rudimentary or completely missing (Figure 4E, arrows). Single interventions by sActRIIB treatment or DR only partially rescued this phenotype. Only in the double intervention group, regularly shaped junctional foldings were observed (Figure 4E, arrows).

These results show that the dual intervention improves muscle physiology over the DR treatment possibly through preserving myofibrillar and NMJ organization.

### Effects of sActRIIB and dietary restriction on organ systems beyond muscle

Beyond muscle directly influenced by attenuation of myostatin/activin signalling, we also assessed some other organs potentially indirectly affected. As motor neurons also contribute to age‐associated loss of muscle function, and *Ercc1* ^Δ/−^ mice show a clear motor neuron degenerative phenotype,^11^ we next examined parameters of genotoxic stress, astrocytosis, and microgliosis in the spinal cords of these animals. Both the number of genotoxic‐responsive transcription factor p53‐positive cells, and the area with increased immunoreactivity of GFAP (astrocytes), IBA1 (microglia), and Mac2 (phagocytosing microglia) were remarkably increased in *Ercc1* ^Δ/−^ compared with WT mice (Figure S5A–D). Only DR was able to significantly reduce the observed microgliosis, while no obvious differences were noted by sActRIIB treatment (Figure S5).

**Figure 5:**
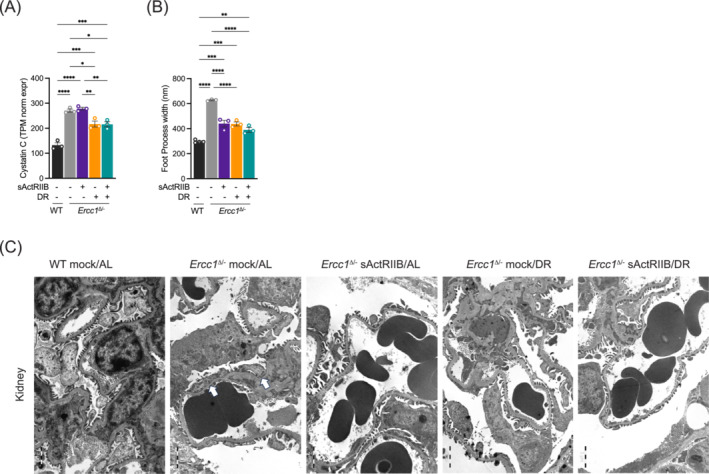
Impact of dual intervention on *Ercc1* ^Δ/−^ kidney function and ultrastructure. (A) In *Ercc1* ^Δ/−^ kidney the expression of cystatin C doubled as compared with WT mice. DR alone or in combination with sActRIIB‐treatment partly rescued this phenotype while sActRIIB‐treatment alone does not. (B, C) As a morphological correlate for the functional alteration the renal filtration barrier of *Ercc1* ^Δ/−^ mice displays a significant foot process effacement of about 600 nm (B, C, arrows), which is partly rescued by sActRIIB‐treatment or dietary restriction alone as well as by the combination of both interventions. *n* = 3 per intervention. *P < 0.05, **P < 0.01, ***P < 0.001, ****P < 0.0001.

Next, we assessed glomerular anomalies as *Ercc1* ^Δ/−^ kidneys were previously found improved by both interventions individually.^12^, ^16^, ^22^, ^26^, ^27^, ^28^ Morphological signs of kidney dysfunction, based on haematoxylin‐eosin‐staining, were virtually absent in WT and elevated in *Ercc1* ^Δ/−^ mice (Figure S6A,B). Although no substantial morphological improvements were noted by any of the interventions (Figure S6C–H), Cystatin C expression levels, indicative of poor kidney functioning, were elevated in *Ercc1* ^Δ/−^ mice compared with WT and partially restored in mice subjected to DR (Figure 5A). At the ultrastructural level, we observed a mild but significant foot process effacement (FPE), indicative of reduced podocyte functioning, in untreated *Ercc1* ^Δ/−^ kidneys (Figure 5B,C). Both sActRIIB and DR restored the foot process width (Figure 5B,C), which was maintained in the dual intervention cohort.

These results show that both interventions partially restored renal ultrastructure and function, with no additional effect from the combination treatment.

### Transcriptomic responses triggered by sActRIIB and dietary restriction in muscle and kidney

Previously, we and others found that age‐related DNA damage accumulation results in a gene‐length‐dependent transcription decline across many tissues and organisms and that DR could partially alleviate this.^12^, ^13^, ^29^, ^30^ To further explore the differential effects of DR, sActRIIB, and combined treatment and potential differences of how organs respond to these interventions, we compared the transcriptomes of quadriceps muscle and kidney in 16‐week‐old mice. Transcriptome analysis of AL‐fed mock‐treated *Ercc1* ^Δ/−^ muscle and kidney revealed a clear separation from WT by principal component analysis (PCA) (Figure S7A,B). Consistent with data from *Ercc1* ^Δ/−^ liver,^12^ both organs showed a preferential down‐regulation of long genes (Figure S7C,D). The top 500 longest expressed genes were mostly downregulated (Figure S7C), being more pronounced in kidney, while the ratio of up:down genes declined with gene length (Figure S7D). This observation of a selective gene‐length‐dependent decline in transcription is fully consistent with the stochastic accumulation of (transcription‐blocking) DNA lesions affecting longer genes more than shorter genes, also known as transcription stress, and was observed before in other tissues during normal and accelerated ageing.^12^, ^13^, ^30^, ^31^, ^32^ In both organs differentially expressed genes (DEGs; FDR < 0.05 and LogFC>|0.5|) were primarily enriched in upstream regulators TP53, TGFβ, TNFα, and INFγ and related signalling processes like p53 signalling and cell cycle: G2/M DNA damage checkpoint regulation. Additional changes specifically triggered in muscle were more related to Foxo and MyoD signalling while in kidney to IL1 (Figure S7E–J).

**Figure 6:**
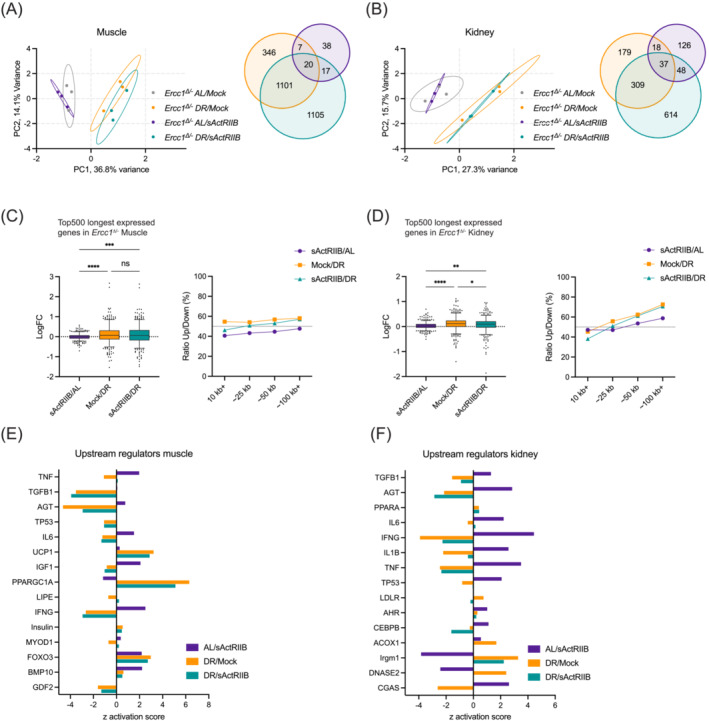
Transcriptome analysis of the various interventions in muscle and kidney by PCA, gene lengths, and upstream regulators. (A, B) Principal component analysis (PCA; left) and Venn diagrams of differently expressed genes (DEGs; right) in quad muscle (A) and kidney (B) of 16‐week‐old *Ercc1* ^Δ/−^ mice subjected to sActRIIB or mock injections and under DR or AL feeding regimens. (C, D) Mean log fold‐change of the 500 longest expressed genes (left) and the ratio up:down within bins of 500 expressed genes across various gene length categories (right) for muscle (C) and kidney (D). Whiskers indicate 5–95 percentile. (E, F) Upstream regulator analysis of the effects of sActRIIB, DR, and double intervention in both organs. *P < 0.05, **P < 0.01, ***P < 0.001, ****P < 0.0001.

Further transcriptome comparison of the four *Ercc1* ^Δ/−^ treatment groups (sActRIIB/AL, Mock/DR, and sActRIIB/DR versus Mock/AL) showed that muscle transcriptome profiles were clearly separated by DR and to a lesser extent by sActRIIB using PCA (Figure 6A). Accordingly, expression changes in the DR‐treated animals were more pronounced (1474 DEGs identified for Mock/DR and 2243 for sActRIIB/DR) compared with sActRIIB‐treatment alone (82 DEGs) (Figure 6A). DR yielded an about equal distribution between up‐ and downregulated DEGs, except for plasma membrane and extracellular matrix associated genes which were relatively more downregulated. The few DEGs induced by sActRIIB were mostly upregulated (Figure S8A–C). In kidney, DR also resulted in strong separation of transcriptome profiles from AL in PCA plots, with again more DEGs identified following DR (543 DEGs for DR and 1008 for sActRIIB/DR) over sActRIIB alone (229 DEGs). As in muscle, plasma membrane and extracellular matrix associated DEGs were more downregulated by DR and all types of DEGs more upregulated by sActRIIB (Figures 6B and S8D–F). However, even though sActRIIB relatively triggers more changes in kidney compared with muscle, no clear separation on PCA was noted (Figure 6B).

In view of the preferential reduced expression of long genes in Mock/AL *Ercc1* ^Δ/−^ muscle and kidney (Figure S7C,D) and the attenuation of this phenomenon in *Ercc1* ^Δ/−^ liver by DR,^12^, ^30^, ^33^ we determined the directional changes of the top 500 longest expressed genes and the ratio of up:down genes across gene length categories. This analysis indicated a mild increase in long gene expression in both organs by DR (Figure 6C,D), pointing to a partial rescue of gene‐length‐dependent transcriptional decline by DR in these tissues. The gene‐length‐dependent re‐expression of primarily longer genes by DR was mirrored by the double treatment but not sActRIIB alone (Figure 6C,D), suggestive of reduced levels of damage by lowering food intake. Consistently, upstream regulator analysis revealed a clear reduction of TP53 and pro‐inflammatory markers IL1B, IL6, IFNγ, and TNFα by DR in both organs (Figure 6E,F), in line with corresponding pathway changes, reduced senescence, and activated anti‐oxidant protective responses and autophagy (Figures S8G–J and S9A–D). Also, tissue homeostasis related factors TGFβ and AGT were activated by sActRIIB but suppressed by DR or double treatment. Specifically in muscle we noted an anti‐correlation between IGF1, Insulin, and UCP1, with suppression of IGF1 and activation of Insulin and UCP1 by DR. Additionally, MyoD and BMP10 were mostly activated by sActRIIB. In kidney, responses were more scattered with a clear activation of CGAS‐pathway by sActRIIB, and suppression by DR, yielding a net null effect by the double treatment.

In summary, DR and the double intervention alleviate transcription‐blocking damage in both organs thereby restoring the expression of many genes, improving organ functioning and boosting overall health, while sActRIIB could be seen more as intervention preserving muscle, blocking its natural wasting route, and functioning mainly along the TGFb‐BMP‐Foxo axis rather than activating multiple signalling pathways.

## Discussion

In this study, we present data showing the effect of combining a regime that suppresses growth and extends lifespan (DR) with one that boosts growth and compresses morbidity (inhibition of myostatin/activin‐signalling). Here we report that the amalgamation of these approaches brings about an extension of both lifespan and health‐span. Our studies here on the lifespan of progeroid DNA repair‐deficient *Ercc1* ^Δ/−^ mice confirmed our previous finding that the inhibition of myostatin/activin signalling (injection of sActRIIB) had no impact on when the mice died (approximately 20–24 weeks). However, when this intervention was given together with DR, the animals were able to live as long if not longer than the DR regime alone. One parsimonious interpretation of these results could be that sActRIIB had little effect when administered with DR. However, this was evidently not the case after monitoring of body weights; mice on the dual regime were heavier than those on DR alone. Furthermore, we showed that the increase in body mass could be attributed in large part to growth of muscle. Most of the other organ masses (heart, kidney, brain, liver, spleen) were unaffected following the introduction of sActRIIB to DR, concluding that the sActRIIB‐regime had a biological impact in the DR progeroid mice. It is interesting to note that within the limits of the low food amounts, since the sActRIIB+DR mice received the same amount of food as the DR‐mock mice, the animals can still influence the fraction for increased body mass. Most importantly we show that the dual intervention resulted in significant physiological improvements (for further details, see the supporting information).

By comparing the outcomes of single versus dual interventions, our studies highlight differing responses in the two key organs examined. We show a synergistic effect of DR and sActRIIB in muscle. However, in kidney, we show beneficial effects of either intervention but no further advantage when combined. Crucially, the dual treatments were not injurious. Hence, our studies show the safety and feasibility of the interventions and that some organs benefit more than others under DR and sActRIIB treatment.

One of the central tenets for the development of ageing features is the allocation of resources between pathways that control maintenance and those regulating growth (mediated in large part by IGF1 signalling). As the challenge of dealing with ageing increases over time, the organism needs to funnel more resources towards maintenance at the expense of growth.^7^, ^34^ Therefore, tissue/organ growth should impact negatively organismal health in the elderly. Attenuation of IGF1 signalling leading to extended lifespan is a feature in numerous animal models.^35^ Furthermore, studies of patients with Acromegaly, all of whom displayed elevated levels of circulating IGF1, have poor quality of health and life in old age.^36^ Even in non‐ageing context there are numerous examples, especially from the livestock industry where the exaggerated growth of one tissue (particularly muscle) comes at the expense of others and usually to the detriment of the organism. For example, broiler chickens developed for extremely large breast muscle have smaller hearts and lungs compared with normal chickens.^37^ However, in this study, we show that promoting muscle growth even in the context of DR had positive outcomes not only in tissue architecture but also functionality. Furthermore, we show that this outcome can be delivered, not by inducing an exogenous signalling programme but instead by attenuating the activity of an innate process (myostatin/activin‐pathway). Hence, a major issue that needs to be addressed is why have the progeric mice activated a programme that hinders healthier ageing? We suggest that clues to answering this question may arise when viewing the allocation of resources, not only to deal with challenges that the animals face presently but also ones that they may encounter in the future. This has been contextualized in the field of farm animal production as the Barrel Model first proposed by Weiner^38^ and later modified by Huber.^39^ The central to the model is a vessel that hold all the organisms' resources (the barrel), fed by a series of funnels that deliver nutrients. The barrel itself feeds several biological processes (maintenance, ontogenic growth, production, and reproduction) through taps. At the stage of adult life, ontogenic growth can be discounted and so is not important in this discussion. During ageing, as outlined above, an organism expends increasing resources to maintenance, so the rate of emptying is elevated compared with earlier stages of life. Crucially, the tap for production (growth of a tissue) is at a lower level than the one for reproduction. Therefore, according to this model, allocation resources to growth results in an emptying of the barrel to levels where resources cannot be delivered for reproduction. We suggest that the myostatin/activin‐signalling is a mechanism that regulates resource allocation towards reproduction since it is key in the development of testis and ovaries.^40^ Hence, we suggest that while it is possible to include muscle growth even during a phase where extra resources are required for maintenance, this comes at an expense to the reproduction system and ultimately the survival of the animal's lineage. Indeed, we show that the dual intervention resulted in smaller testis compared with either single intervention. Future studies are planned to investigate this hypothesis by examining fertility rates of mice on the differing interventions described here.

Most of the data described thus far comes from studies where the interventions were started at the onset of ageing phenotypes in the progeroid model. One other significant finding of this study emerged when we determined the impact of rolling out sActRIIB‐treatment at a time when the progeroid phenotype was quite advanced (e.g., when all mice normally had severe tremors). Here we found that late commencement of sActRIIB to DR was not only tolerated by the mice but that the mice had lifespans far greater than sActRIIB alone. Furthermore, these mice failed to develop any form of tremors (as seen with DR alone). Unexpectedly, we found that the late implementation of sActRIIB‐treatment resulted in total normalization of imbalance problems. Herein, we show that all progeric mice displayed issues with balancing by week 20, whereas the DR treated mice reached this time point at week 40. However, late implementation of sActRIIB resulted in none of the mice developing issues related to balance. The mechanisms underpinning the benefits of late myostatin/activin neutralization will be explored in depth in future studies. However, these studies show from a health perspective that promoting muscle growth does not have to be started before signs of ageing for it to reap benefits, as long as there has been a degree of calorie control.

## Conflict of interest

The authors declare no competing interests.

## Supporting information


**Table S1.** Antibody details

**Figure S1.** Sex segregation of whole body data (A‐B) Mean body weights development (±SD) of *Ercc1* ^Δ/−^ mice under sActRIIB and/or dietary restriction (DR) conditions versus mock‐treated *ad libitum* (AL) fed separated by gender. All treatments were initiated from 8 weeks of age. All groups consist of 3 females (A) and 3 males (B). (C‐D) Food intake (C) and AUC thereof (D) of the total mixed gender cohort (*n* = 3 females + 3 males). (E‐F) Survival data of Figure 1C separated by gender. (G‐I) Onset of neurological abnormalities tremors, severe tremors, and imbalance with age under AL and DR conditions when sActRIIB was administered late in life from 16 weeks of age while the DR intervention was still initiated from 8 weeks of age. ****P < 0.0001
**Figure S2.** Exercise profiling and muscle weights of *Ercc1* ^Δ/−^ mice. (A‐B) Grip strength measure of forelimbs (A) and all limbs (B) in Newton. (C‐G) Dissected muscle weights for tibialis anterior (TA), extensor digitorum longus (EDL), gastrocnemius (Gas.), soleus (Sol.), and plantaris (Plant.), and (H‐L) normalisation of key muscle weights to body weigth. All measures from 16‐week‐old male mice. *n* = 4 males per group. *P < 0.05, **P < 0.01, ***P < 0.001, ****P < 0.0001.
**Figure S3.** Organ and blood profiling of *Ercc1* ^Δ/−^ mice. (A) Measure of Tibial length. (B‐E) Normalisation of Liver (B), Kidney (C), Brain (D), and Spleen (E) to body weight. (F‐J) Weights of heart (F), lung (G), stomach (H), testis (I) and thymus (J) and (K‐O) normalisation of these organs to body weight. Measure of (P) blood glucose, (Q) cholesterol, and (R) triglycerides. All measures from 16‐week‐old male mice. *n* = 4 males per group. *P < 0.05, **P < 0.01, ***P < 0.001, ****P < 0.0001.
**Figure S4.** Profiling of *Ercc1* ^Δ/−^ EDL muscle fibre size and MHC distribution. (A) EDL muscle fibre size profiling based on MHC expression. (B) EDL MHC composition based on MHC expression. EDL muscles from 16 week old mice. Between 55–75 fibres were counted from each mouse before being averaged per cohort. *n* = 8 WT Mock/AL, *n* = 7/8 *Ercc1* ^Δ/−^ Mock/AL, n = 7/8 *Ercc1* ^Δ/−^ sActRIIB/AL, n = 7/8 *Ercc1* ^Δ/−^ Mock/DR, n = 7/8 *Ercc1* ^Δ/−^ sActRIIB/DR. *P < 0.05, **P < 0.01, ***P < 0.001, ****P < 0.0001.
**Figure S5.** p53, GFAP, IBA1 and Mac2 quantification. Quantification of the number of p53‐positive cells in spinal cord (A) and relative intensity of spinal cord sections immunoperoxidase‐stained for GFAP (B), IBA1 (C), and Mac2 (D). *P < 0.05, **P < 0.01, ***P < 0.001, ****P < 0.0001.
**Figure S6.** Kidney H and E images. Representative pictures of haematoxylin‐eosin‐stained slides from kidney of Mock/AL (A‐B), sActRIIB/AL (C‐D), Mock/DR (E‐F), and sActRIIB/DR (G‐H) treated *Ercc1* ^Δ/−^ mice. Scale bar in A, C, E, and G = 100 um, and in B, D, F, and H 50 um.
**Figure S7.** Gene expression changes induced by DNA repair‐deficiency. (A‐B) Principal component analysis (PCA) of all genes from NGS RNA expression profiles of quadriceps muscle (A) and kidney (B) of untreated 16‐week‐old *Ercc1* ^Δ/−^ and WT mice. (C‐D) Mean log fold‐change of the 500 longest expressed genes with 5–95 percentile (C) and the ratio up:down within different bins of 500 expressed genes of certain gene length classes (D) in *Ercc1* ^Δ/−^ mice versus WT controls for both organs. (E‐F) Gene Ontology (GO) annotated subcellular localization of differentially expressed genes (DEGs) between *Ercc1* ^Δ/−^ and WT depicted for muscle (E) and kidney (F). The numbers of respectively up‐ (red) and down‐regulated (blue) DEGs are indicated in each bar. PM = plasma membrane; ECM = extracellular matrix; Cyto = cytoplasm; Nucl = nucleus. (G‐H) Top 10 most significantly enriched upstream regulators in muscle (G) and kidney (H) based on DEGs, filtered for genes, RNAs and proteins and ordered on p‐value with most significant on top, as identified with Ingenuity Pathway Analysis (IPA). Length of bars indicate the activation z‐score with red indicating upregulated and blue downregulated. (I) Numbers and overlap of DEGs identified between *Ercc1* ^Δ/−^ muscle and kidney. (J) Top 5 significantly altered signalling pathways as identified with IPA for the 611 muscle‐specific DEGs (top), 5008 kidney‐specific DEGs (bottom) and 495 common DEGs (middle), presumably driven by DNA damage accumulation. Length of bars indicate the ‐log10(P‐value). ****P < 0.0001.
**Figure S8.** DEG distribution, muscle and kidney signalling pathways. Gene Ontology (GO) annotated subcellular localization (A‐F) and Ingenuity Pathway Analysis of enriched signalling (G‐H) and metabolic (I‐J) pathways of differentially expressed genes (DEGs) in *Ercc1* ^Δ/−^ muscle (A‐C, G, I) and kidney (D‐F, H, J) of sActRIIB, DR, and double intervention.
**Figure S9.** Heatmaps of selected processes. LogFC values of acetylcholine receptor changes in muscle (A), and cellular senescence (B), anti‐oxidant response (C), and UPR/autophagy (D) related genes in both muscle and kidney depicted for *Ercc1* ^Δ/−^ vs WT, and sActRIIB, DR, and double intervention in *Ercc1* ^Δ/−^ mice versus Mock/AL.
