# Supplementary material for: Improved health by combining dietary restriction and promoting muscle growth in DNA repair‐deficient progeroid mice
Source: J Cachexia Sarcopenia Muscle. 2024 Sep 8;15(6):2361–74. doi: 10.1002/jcsm.13570 (PMC11634475; doi:10.1002/jcsm.13570)
Supplement: Supplementary file 1 — Table S1. Antibody details [file JCSM-15-2361-s001.docx]

**Supplementary Extended Methods**

***Ethic statements***

Animal experiments were performed according to institutional guidelines as overseen by the Animal Welfare Board of the Erasmus MC, following Dutch and EU legislation. Prior to the start of the experiments, a project license for the animal experiments performed for this study was obtained from the Dutch national authority and filed under DEC no. 139-12-13, 139-12-18, and 18-6886-05.

***Mouse model***

The generation and characterization of *Ercc1*^∆/+^ and *Ercc1*^+/−^ mice have been previously described^1,2^. *Ercc1*^∆/−^ mice were obtained by crossing *Ercc1*^∆/+^ (in a pure C57BL6J or FVB background) with *Ercc1*^+/−^ mice (in a pure FVB or C57BL6J background respectively) to yield *Ercc1*^∆/−^ offspring with a genetically uniform F1 C57BL6J/FVB hybrid background. Typical unfavourable characteristics, such as blindness in an FVB background or deafness in a C57BL6J background, do not occur in this hybrid background. Wild-type F1 littermates were used as controls. Hence, all animals used in the studies described here were of the same F1 C57BL6J/FVB hybrid background.

***Housing conditions***

All animals were randomly divided over the various intervention groups and housed in individual ventilated cages under specific pathogen-free conditions. The environment was maintained at a temperature of 20–22°C with 12h light:12h dark cycles. Mice were visually inspected daily for moribund states (or serious illnesses exceeding the common ageing trajectories of the mouse model used). Animals were bred and maintained on AIN93G synthetic pellets (Research Diet Services B.V., Wijk bij Duurstede, Netherlands; gross energy content 4.9 kcal/g dry mass, digestible energy 3.97 kcal/g). Since the *Ercc1*^Δ/−^ mice are smaller, food was administered within the cages and water bottles with long nozzles were used from around 2 weeks of age. Food and water were offered *ad libitum* (AL) unless stated otherwise.

***Interventions***

The amount of DR was determined in a prior pilot study where food intake of the AL-fed mice was continuously monitored^1^. Mice on average ate 2.3 g food per day, resulting in 1.6 g/day for 30% DR, which was kept constant throughout the experiment. Water was freely available. DR was initiated at 7 weeks of age with 10% food reduction, and food was gradually reduced to 30% DR from 9 weeks of age onward as previously published^1^. Food was given to the animals just before the start of the dark (active) period (Zeitgeber Time; ZT12). Our DR regimen in a room with altered day-night rhythm was specifically chosen not to disturb the biological clock, as this may influence the anti-ageing effect of DR.

Post-natal myostatin/activin block was induced in 8-week-old mice, through intraperitoneal (IP) injection with 10 mg/kg of sActRIIB-Fc twice every week as previously published^3^. An additional AL and DR group was subjected to sActRIIB from 16 weeks of age. Effects on body weight changes, lifespan and onset of neurological parameters was assessed in both males and females in a 50/50 ratio, while the additional more detailed examination at 16 weeks of age was performed on males only. End-of-life *Ercc1*^Δ/−^ animals, both sActRIIB and DR treated, were post-mortem investigated and scored negative for visible tumours, signs of internal bleedings, enlarged spleen size, or abnormally coloured heart or enlarged heart size.

***Phenotype scoring and behavioural assays***

The mice were weighed and visually inspected weekly and were scored in a blinded manner by two experienced research technicians for the onset of various phenotypical parameters. Whole-body tremor was scored if mice were trembling for a combined total of 5-10 s when put on a flat surface for 20 s. Tremors were considered severe when the combined total time of trembling was 10-20 s. Impaired balance was determined by observing the mice walking on a flat surface for 20 s. Mice that had difficulties in maintaining an upright orientation during this period were scored as having imbalance. Rotarod performance was assessed by measuring the aver- age time spent on an accelerating rotarod (Ugo Basile). All animals were given four consecutive trials of a maximum of 5 min with inter-trial intervals of 1 h. Grip strength was determined by placing mice with forelimbs or all limbs on a grid attached to a force gauge, and steadily pulling the mice by their tail. Grip strength is defined as the maximum strength produced by the mouse before releasing the grid. For each value the test was performed in duplicate. The balance beam consisted of 1-meter-long and 6 or 12 mm diameter horizontal beam, supported by a metal pole on one end and home cage on the other end. In the training sessions animals learned how to cross the beam from the platform to the cage. Test runs were recorded on video, for assessment of crossing time and the number of slips per run.

***Muscle Histology and Immunohistochemistry***

Muscles were washed once with PBS after dissection and then cleared of excess liquid by dabbing briefly onto a tissue paper, weighed and then placed onto aluminium foil which was then positioned onto the surface of liquid nitrogen frozen isopentane. Cryosectioning was performed to generate 10um thick tissue slices. For immunohistochemistry, frozen sections were equilibrated to room temperature before the application of block solution (5% v/v Foetal Calf Serum, 0.05% v/v Triton X-100 in PBS) for 30 minutes. All antibodies were prepared in block solution and then applied overnight in darkened conditions. Thereafter, sections were washed three times for 5 minutes periods in block solutions before applying secondary antibodies (prepared in block solution) for 1h. Finally, sections were again washed as above before mounting in DAKO mounting media containing DAPI. Details of the primary and secondary antibodies used in this study are given in Table S1.

***Dihydroethidium (DHE) staining***

Air dried tissue sections were rehydrated in PBS for 30 minutes before being overlaid with DHE (50uM made in PBS Sigma D7008) for 30 minutes at 37^o^C in the dark. Thereafter, slides were washed three times in PBS before applying DAKO mounting media containing DAPI.

***SDH staining***

Sections were washed three times in PBS as above before submerging in Sodium Phosphate buffered solution containing 75mM Sodium Succinate, 1.1mM Nitroblue Tetrazolium, 1.03mM Phenazine Methosulphate (all chemicals from Sigma-Aldrich) for a period of 30 minutes at RT. Thereafter, sections were fixed in 10% formal calcium, dehydrated and cleared (in xylene) before the application of DPX mounting media (Fisher).

The images were captured and then converted to 8-bit grayscale, which reduces the colour information to a range of shades from black (least intense) to white (most intense). Using this grayscale representation, we applied a binary threshold to segment the image into two categories: SDH-positive (SDH+) and SDH-negative (SDH-) fibres. The threshold was set with a lower limit of 50 and an upper limit of 130. Pixels within this intensity range were identified as SDH+, indicating the presence of enzyme activity, and were pseudo-coloured red for visual representation. Pixels outside this range were classified as SDH-, indicating minimal or no enzyme activity.

***Muscle Tension Measurements***

After dissection, EDL muscles were kept in oxygenated Kreb’s solution (95% O_2_, 5% CO_2_) which was maintained at 25^o^C. The muscle was tied at both ends with suture wire and the distal end affixed to a hook and the proximal end to the lever of the muscle test system (1200A Muscle Test System, Aurora Scientific, Ireland). Muscles were equilibrated in Kreb’s solution for 10 minutes. Optimal length was determined by applying a fixed voltage stimulation across electrodes placed either side of the muscle. Voltage was then increased to determine the maximum twitch tension. Thereafter, contractions were induced by applying voltage for 500ms at the following frequencies: 20, 50, 100 and 200 Hz.

***Transmission electron microscopy***

For electron microscopy small pieces (2 mm^3^) of mouse renal cortex and biceps muscle (n=3 per group) were immersion fixed in 4% PFA in 0.1 M PB (48 h at 4°C). Tissue blocks were washed in 0.1 M PB, treated with OsO_4_ (0.5% for 45 min at RT) and stained with uranyl acetate (1% w/v in 70% v/v Ethanol, 45 minutes at RT). After dehydration tissue blocks were embedded in epoxy resin (Durcupan ACM, Sigma-Aldrich, Gillingham, UK). 50 nm ultrathin sections were cut using an UC6 ultramicrotome (Leica, Wetzlar, Germany) and analyzed with a 80 kV Philipps CM100 transmission electron microscope and Olympus ITEM software.

***RNA-seq***

RNA from quadriceps muscle and kidneys from *Ercc1*^Δ/−^ and WT animals treated with DR and/or sActRIIB was used from transcriptome analysis. RNA was isolated using the miRNeasy mini kit (QIAgen; 217004) according to manufacturer’s protocol. RNA quality and quantity were assessed using the NanoDrop One and Bioanalyzer 2100 (Agilent, Santa Clara, CA, USA; G2939BA), respectively. Samples were sent for transcriptome sequencing (Novogene, Cambridge, United Kingdom) on the Illumina NovaSeq 6000 platform. Analysis of raw data files was performed on our in house-generated data analysis pipeline. Removal of sequence adaptors from sequence reads was performed using Trimmomatic (version 0.39). Trimmed reads were aligned to mouse reference genome (annotation: gencode.vM20.annotation.gtf; genome: GRCm38.p6.genome.fa; http://gencodegenes.org/mouse/release_M20.html) using STAR (version 2.7.0f). Read counts for each gene were obtained using FeatureCounts (as part of SubRead version 1.6.4) followed by quantification of log fold changes and false discovery rates using EdgeR (version 3.32.1). Ingenuity Pathway Analysis (QIAgen, version 81348237) was used for subcellular distribution, signalling and metabolic pathway analyses and identification of potentially changed upstream regulators. TPM normalized reads from WT animals were used to determine genes expressed in muscle or kidney respectively and ordered based on gene length. Mean LogFC and ratio up:down was calculated form the different interventions in *Ercc1*^Δ/−^ mice for the 500 longest genes or different bins on 500 genes of certain length categories. All data files have been submitted to the NCBI gene expression omnibus (GEO) under GSE268971.

***Statistical analysis***

Statistical analyses were performed using GraphPad Prism Software (San Diego, CA, United States, version 10.1.0). One-way ANOVA with Tukey’s multiple comparison was used for analyses between genotype and interventions with multiple groups except muscle fibre area and fibre profile for which we used two-way ANOVA. Area under the curve (AUC) for bodyweight and food intake was calculated as the value of log10 between indicated weeks. Log-rank (Mantel-Cox) tests were used to compare curves for onset of tremors, imbalance and survival. Hazard plot shows log values of hazard ratio (logrank), when compared to control group and 95% confidence interval. Graphs illustrate individual values, means and standard error unless otherwise indicated. *P < 0.05, **P < 0.01, ***P < 0.001, ****P < 0.0001.

**Supplementary Discussion**

We found the physiological improvements observed to manifest in key functional deficits developing much later in the dual intervention compared with dietary restriction (DR) alone (e.g., development of imbalance impairment) as well as improvements in functional readout of physiological activities. Whole animal studies showed beneficial effects of the dual intervention on latency to fall measures as well as balance beam performance. Furthermore, the *ex vivo* examination of muscle function showed that specific force significantly increased in the dual intervention cohort compared to DR alone. Indeed, that level of specific force in the dual intervention cohort was the same as the sActRIIB regime alone. The improvements in latency to fall and balance tests cannot be solely attributed to increases in muscle mass in the dual intervention cohort. These activities rely on the action of muscle and motor nerves. We provide ultrastructural evidence that the dual intervention alone was able to bring about beneficial changes at the key interface between muscle and nerve, namely the neuromuscular junction (NMJ). Numerous studies have shown that molecular interventions limited to skeletal muscle alone (hence not systemic or involving non-skeletal muscle) that induce change in mass are accompanied by changes in the NMJ (e.g**.,**^4^).

Given the profound effect of the sActRIIB intervention on both *ad libitum* (AL) fed *Ercc1*^Δ/-^ mice or *Ercc1*^Δ/-^ mice on a DR regime it was very surprising that the molecular landscape was relatively unaffected by the sActRIIB treatment. Our principal component analysis examination showed that sActRIIB failed to give a clear segregation outcome when applied to either cohorts. Indeed, this was also found after the examination of the kidney transcriptome. We suggest a number of possible explanations for the incongruence between tissue structure/function and molecular profile. Previously, we hypothesized that DNA damage occurs stochastically throughout the genome and thus longer genes would have a higher likelihood of containing a damage compared to shorter genes^1^. And if such lesion would impede transcription, such as a bulky adduct, this would overall result in more longer genes to be down regulated (transcription stress; see also explanation in^5^), indicative of more transcription-blocking DNA lesions (TBLs). Indeed, with increasing damage we found more long genes to be downregulated in both *Ercc1*^Δ/-^ mouse liver and WT rat liver and even in the ageing human brain^1^. Importantly, this was recently confirmed as a consistent phenomenon of ageing across many organs and species ranging from worms to mammals^6-9^. More importantly, this measure of TBL-damage was significantly lowered by DR in *Ercc1*^Δ/-^ deficient mice, in which normally higher levels of transcription stress are present in at least liver^1^, while for example rapamycin, a commonly proposed DR mimetic, did not^10^. Here we show that this phenomenon is also happening in *Ercc1*^Δ/-^ muscle and kidney. Upon treatment with DR alone or the combination of sActRIIB+DR we found the opposite, that longer genes were again more upregulated compared to shorter genes. This indicates that DR and the double intervention alleviate transcription stress in both organs and thus restoring the expression of many genes. These findings are further strengthened by the reduced upstream regulator analysis of TP53 for DR and double intervention and match with the remarkable lifespan extension observed with these two interventions. Moreover, we found markers of cellular senescence in *Ercc1*^Δ/-^ muscle and kidney to be dampened by DR and double intervention, while UPR and autophagy components seemed more activated by these treatments, in accordance with lower levels of damage and improved health. sActRIIB did not normalize *Ercc1*^Δ/-^ muscle and kidney gene expression in a length-dependent manner. The slightly increased longest transcript in sActRIIB kidney could likely be a result of increased cell proliferation, in line with the further activation of the DNA damage response sensor TP53 and subsequent pro-inflammatory factors. Also, it is possible that there are master regulators of tissue development rather than those required for wholesale modification of the entire transcriptome. In this way sActRIIB could be seen more as intervention preserving muscle, blocking its natural wasting route, and functioning mainly along the TGFb-BMP-Foxo axis rather than activating multiple signalling pathways.

Against the background of steadily increasing life expectancy, the known decline in kidney function and the increased susceptibility of the kidneys to damage in old age is becoming increasingly relevant both medically and economically^11,12^. A key role in the ageing process of the kidney is played by the glomerular renal filter and, in particular, by the specialized epithelium of the glomerulus, the podocytes. Since podocytes influence all other glomerular cell types in a paracrine manner^13^, age-related damage or loss of podocytes trigger pathophysiological cascades that ultimately lead to glomerulosclerosis. Thus, ageing of the glomerular filter can be considered as a podocytopathy^14-16^. As one of the hallmarks of damaged and/or ageing podocytes^17^, we found a significant podocyte foot process effacement (FPE) in untreated *Ercc1*^Δ/-^ mice. This was in line with our previous findings where we investigated the impact of sActRIIB on renal morphology and function^3^. As described there, sActRIIB was also able to restore FPE in the present study. The same applies to DR and the combination of both interventions, although no additive effect was observed. Asking for the underlying molecular mechanisms the examination of the kidney transcriptome of *Ercc1*^Δ/-^ mice displayed some well-known alterations described in the ageing kidney and/or podocytes^18^. Namely we observed an up-regulation of senescence and pro-inflammatory genes e.g., CDKN1A&2A, IL1a, IL6 and TNFa. In contrast, untreated *Ercc1*^Δ/-^ mice displayed a significant down-regulation of pro-autophagy genes. Both single interventions could restore these alterations whereby DR was more effective in both cases. Since podocytes are highly dependent on a proper autophagic flux^19,20^ and the accumulation of senescent cells has been shown to be associated with age-related glomerulosclerosis and decline in renal function^21-23^ the restoration of these pathways by both interventions may at least partly explain their beneficial effect on podocyte morphology observed here. Whether the effect of sActRIIB treatment on podocytes is direct or is mediated via other renal cells that have been shown to possess the activin receptor^24^, remains to be seen. In a murine model of chronic kidney disease, we were recently able to show that interorgan signalling between the kidney and muscle and *vice versa* also has a significant influence on pathophysiological processes in both organs^24^. Further studies must show whether this also applies to age-related changes. Beneficial effects of DR on renal ageing have already been shown in the rat model, e.g. DR slows down the age-related loss of podocytes and prevents podocyte hypertrophy^25^. As in the present study, the underlying molecular signalling pathways are unknown. Possible candidates are sirtuins, a family of nicotinamide adenine dinucleotide (NAD)–dependent deacetylases which are on the one hand effectors of DR and on the other hand have beneficial effects on autophagy in podocytes^26,27^. Indeed, enhancing NAD levels has been shown to boost health and extend lifespan^28^, recently also in *Ercc1*^Δ/-^ mice^29^. However, since podocytes represent a rather small population of cells within the kidney RNA-seq of the whole organ for sure has its limitations in detecting podocyte specific pathways. New approaches like single nuclei RNA-seq in *Ercc1*^Δ/-^ kidneys are needed to dissect the contribution of the various renal cell types to kidney ageing and to identify druggable cell specific pathways which can be used to mitigate renal ageing.

**Supplementary Figure Legends**

**Figure S1. Sex segregation of whole body data**

(A-B) Mean body weights development (±SD) of *Ercc1*^Δ/−^ mice under sActRIIB and/or dietary restriction (DR) conditions versus mock-treated *ad libitum* (AL) fed separated by gender. All treatments were initiated from 8 weeks of age. All groups consist of 3 females (A) and 3 males (B). (C-D) Food intake (C) and AUC thereof (D) of the total mixed gender cohort (n=3 females + 3 males). (E-F) Survival data of Figure 1C separated by gender. (G-I) Onset of neurological abnormalities tremors, severe tremors, and imbalance with age under AL and DR conditions when sActRIIB was administered late in life from 16 weeks of age while the DR intervention was still initiated from 8 weeks of age. ****P<0.0001

**Figure S2. Exercise profiling and muscle weights of *Ercc1*^Δ/−^ mice.**

(A-B) Grip strength measure of forelimbs (A) and all limbs (B) in Newton. (C-G) Dissected muscle weights for tibialis anterior (TA), extensor digitorum longus (EDL), gastrocnemius (Gas.), soleus (Sol.), and plantaris (Plant.), and (H-L) normalisation of key muscle weights to body weigth. All measures from 16-week-old male mice. n=4 males per group. *P<0.05, **P<0.01, ***P<0.001, ****P<0.0001.

**Figure S3. Organ and blood profiling of *Ercc1*^Δ/−^ mice.**

(A) Measure of Tibial length. (B-E) Normalisation of Liver (B), Kidney (C), Brain (D), and Spleen (E) to body weight. (F-J) Weights of heart (F), lung (G), stomach (H), testis (I) and thymus (J) and (K-O) normalisation of these organs to body weight. Measure of (P) blood glucose, (Q) cholesterol, and (R) triglycerides. All measures from 16-week-old male mice. n=4 males per group. *P<0.05, **P<0.01, ***P<0.001, ****P<0.0001.

**Figure S4. Profiling of *Ercc1*^Δ/−^ EDL muscle fibre size and MHC distribution.**

(A) EDL muscle fibre size profiling based on MHC expression. (B) EDL MHC composition based on MHC expression. EDL muscles from 16 week old mice. Between 55-75 fibres were counted from each mouse before being averaged per cohort. n=8 WT Mock/AL, n=7/8 *Ercc1*^Δ/−^ Mock/AL, n=7/8 *Ercc1*^Δ/−^ sActRIIB/AL, n=7/8 *Ercc1*^Δ/−^ Mock/DR, n=7/8 *Ercc1*^Δ/−^ sActRIIB/DR. *P<0.05, **P<0.01, ***P<0.001, ****P<0.0001.

**Figure S5. p53, GFAP, IBA1 and Mac2 quantification**

Quantification of the number of p53-positive cells in spinal cord (A) and relative intensity of spinal cord sections immunoperoxidase-stained for GFAP (B), IBA1 (C), and Mac2 (D). *P<0.05, **P<0.01, ***P<0.001, ****P<0.0001.

**Figure S6. Kidney H and E images**

Representative pictures of haematoxylin-eosin-stained slides from kidney of Mock/AL (A-B), sActRIIB/AL (C-D), Mock/DR (E-F), and sActRIIB/DR (G-H) treated *Ercc1*^Δ/−^ mice. Scale bar in A, C, E, and G = 100 um, and in B, D, F, and H 50 um.

**Figure S7. Gene expression changes induced by DNA repair-deficiency**

(A-B) Principal component analysis (PCA) of all genes from NGS RNA expression profiles of quadriceps muscle (A) and kidney (B) of untreated 16-week-old *Ercc1*^Δ/−^ and WT mice. (C-D) Mean log fold-change of the 500 longest expressed genes with 5-95 percentile (C) and the ratio up:down within different bins of 500 expressed genes of certain gene length classes (D) in *Ercc1*^Δ/−^ mice versus WT controls for both organs. (E-F) Gene Ontology (GO) annotated subcellular localization of differentially expressed genes (DEGs) between *Ercc1*^Δ/−^ and WT depicted for muscle (E) and kidney (F). The numbers of respectively up- (red) and down-regulated (blue) DEGs are indicated in each bar. PM = plasma membrane; ECM = extracellular matrix; Cyto = cytoplasm; Nucl = nucleus. (G-H) Top 10 most significantly enriched upstream regulators in muscle (G) and kidney (H) based on DEGs, filtered for genes, RNAs and proteins and ordered on p-value with most significant on top, as identified with Ingenuity Pathway Analysis (IPA). Length of bars indicate the activation z-score with red indicating upregulated and blue downregulated. (I) Numbers and overlap of DEGs identified between *Ercc1*^Δ/−^ muscle and kidney. (J) Top 5 significantly altered signalling pathways as identified with IPA for the 611 muscle-specific DEGs (top), 5008 kidney-specific DEGs (bottom) and 495 common DEGs (middle), presumably driven by DNA damage accumulation. Length of bars indicate the -log10(P-value). ****P<0.0001.

**Figure S8. DEG distribution, muscle and kidney signalling pathways**

Gene Ontology (GO) annotated subcellular localization (A-F) and Ingenuity Pathway Analysis of enriched signalling (G-H) and metabolic (I-J) pathways of differentially expressed genes (DEGs) in *Ercc1*^Δ/−^ muscle (A-C, G, I) and kidney (D-F, H, J) of sActRIIB, DR, and double intervention.

**Figure S9. Heatmaps of selected processes**

LogFC values of acetylcholine receptor changes in muscle (A), and cellular senescence (B), anti-oxidant response (C), and UPR/autophagy (D) related genes in both muscle and kidney depicted for *Ercc1*^Δ/−^ vs WT, and sActRIIB, DR, and double intervention in *Ercc1*^Δ/−^ mice versus Mock/AL.

**Table S1. Antibody details**

| Antibody | Species | Dilution | Supplier |
| --- | --- | --- | --- |
| MHCIIA | Mouse | 1:1 | DSHB A4.74 |
| MHCIIB | Mouse | 1:1 | DSHB BF.F3 |
| CD31 | Rat | 1:150 | AbD serotec MCA2388 |
| p53 | Rabbit | 1:1000 | Leica; NCL-p53-CMP5, AB_2744683 |
| GFAP | Rabbit | 1:8000 | DAKO; Z0334, AB_10013382 |
| IBA1 | Rabbit | 1:5000 | Wako; 019-19741 |
| Mac2 (LGALS3) | Rat | 1:1000 | Cedarlane; CL8942AP, AB_10060357 |
| Alexa fluor 633 anti-mouse | Goat | 1:200 | Life Technologies # A20146 |
| Alexa fluor 488 anti-mouse | Goat | 1:200 | Life Technologies # A11029 |

**Supplementary References**

1 Vermeij, W. P. *et al.* Restricted diet delays accelerated ageing and genomic stress in DNA-repair-deficient mice. *Nature* **537**, 427-431 (2016).

2 Weeda, G. *et al.* Disruption of mouse ERCC1 results in a novel repair syndrome with growth failure, nuclear abnormalities and senescence. *Curr Biol* **7**, 427-439 (1997).

3 Alyodawi, K. *et al.* Compression of morbidity in a progeroid mouse model through the attenuation of myostatin/activin signalling. *J Cachexia Sarcopenia Muscle* **10**, 662-686 (2019).

4 Fries, J. F. Aging, natural death, and the compression of morbidity. *N Engl J Med* **303**, 130-135 (1980).

5 Lans, H., Hoeijmakers, J. H. J., Vermeulen, W. & Marteijn, J. A. The DNA damage response to transcription stress. *Nat Rev Mol Cell Biol* **20**, 766-784 (2019).

6 Gyenis, A. *et al.* Genome-wide RNA polymerase stalling shapes the transcriptome during aging. *Nat Genet* **55**, 268-279 (2023).

7 Ibañez-Solé, O., Barrio, I. & Izeta, A. Age or lifestyle-induced accumulation of genotoxicity is associated with a length-dependent decrease in gene expression. *iScience* **26**, 106368 (2023).

8 Stoeger, T. *et al.* Aging is associated with a systemic length-associated transcriptome imbalance. *Nat Aging* **2**, 1191-1206 (2022).

9 Soheili-Nezhad, S., van der Linden, R. J., Olde Rikkert, M., Sprooten, E. & Poelmans, G. Long genes are more frequently affected by somatic mutations and show reduced expression in Alzheimer's disease: Implications for disease etiology. *Alzheimers Dement* **17**, 489-499 (2021).

10 Birkisdóttir, M. B. *et al.* Unlike dietary restriction, rapamycin fails to extend lifespan and reduce transcription stress in progeroid DNA repair-deficient mice. *Aging Cell* **20**, e13302 (2021).

11 Jha, V. *et al.* Chronic kidney disease: global dimension and perspectives. *Lancet* **382**, 260-272 (2013).

12 Hommos, M. S., Glassock, R. J. & Rule, A. D. Structural and Functional Changes in Human Kidneys with Healthy Aging. *J Am Soc Nephrol* **28**, 2838-2844 (2017).

13 Dimke, H., Maezawa, Y. & Quaggin, S. E. Crosstalk in glomerular injury and repair. *Curr Opin Nephrol Hypertens* **24**, 231-238 (2015).

14 Wiggins, J. E. Aging in the glomerulus. *J Gerontol A Biol Sci Med Sci* **67**, 1358-1364 (2012).

15 Hodgin, J. B. *et al.* Glomerular Aging and Focal Global Glomerulosclerosis: A Podometric Perspective. *J Am Soc Nephrol* **26**, 3162-3178 (2015).

16 Floege, J. *et al.* Age-related glomerulosclerosis and interstitial fibrosis in Milan normotensive rats: a podocyte disease. *Kidney Int* **51**, 230-243 (1997).

17 Kriz, W., Shirato, I., Nagata, M., LeHir, M. & Lemley, K. V. The podocyte's response to stress: the enigma of foot process effacement. *Am J Physiol Renal Physiol* **304**, F333-347 (2013).

18 Shankland, S. J. *et al.* Podocyte Aging: Why and How Getting Old Matters. *J Am Soc Nephrol* **32**, 2697-2713 (2021).

19 Lenoir, O., Tharaux, P. L. & Huber, T. B. Autophagy in kidney disease and aging: lessons from rodent models. *Kidney Int* **90**, 950-964 (2016).

20 Bechtel, W. *et al.* Vps34 deficiency reveals the importance of endocytosis for podocyte homeostasis. *J Am Soc Nephrol* **24**, 727-743 (2013).

21 van Deursen, J. M. The role of senescent cells in ageing. *Nature* **509**, 439-446 (2014).

22 Zhang, L. *et al.* C/EBPα deficiency in podocytes aggravates podocyte senescence and kidney injury in aging mice. *Cell Death Dis* **10**, 684 (2019).

23 Sweetwyne, M. T. *et al.* The mitochondrial-targeted peptide, SS-31, improves glomerular architecture in mice of advanced age. *Kidney Int* **91**, 1126-1145 (2017).

24 Solagna, F. *et al.* Pro-cachectic factors link experimental and human chronic kidney disease to skeletal muscle wasting programs. *J Clin Invest* **131** (2021).

25 Wiggins, J. E. *et al.* Podocyte hypertrophy, "adaptation," and "decompensation" associated with glomerular enlargement and glomerulosclerosis in the aging rat: prevention by calorie restriction. *J Am Soc Nephrol* **16**, 2953-2966 (2005).

26 Kume, S. *et al.* Calorie restriction enhances cell adaptation to hypoxia through Sirt1-dependent mitochondrial autophagy in mouse aged kidney. *J Clin Invest* **120**, 1043-1055 (2010).

27 Salminen, A. & Kaarniranta, K. SIRT1: regulation of longevity via autophagy. *Cell Signal* **21**, 1356-1360 (2009).

28 Lopez-Otin, C., Galluzzi, L., Freije, J. M. P., Madeo, F. & Kroemer, G. Metabolic Control of Longevity. *Cell* **166**, 802-821 (2016).

29 Birkisdóttir, M. B. *et al.* The use of progeroid DNA repair-deficient mice for assessing anti-aging compounds, illustrating the benefits of nicotinamide riboside. *Frontiers in Aging* **3** (2022).
