# Supplementary material for: Improved health by combining dietary restriction and promoting muscle growth in DNA repair‐deficient progeroid mice
Source: J Cachexia Sarcopenia Muscle. 2024 Sep 8;15(6):2361–74. doi: 10.1002/jcsm.13570 (PMC11634475; doi:10.1002/jcsm.13570)
Supplement: Supplementary file 2 — Figure S1. Sex segregation of whole body data (A‐B) Mean body weights development (±SD) of Ercc1 Δ/− mice under sActRIIB and/or dietary restriction (DR) conditions versus mock‐treated ad libitum (AL) fed separated by gender. All treatments were initiated from 8 weeks of age. All groups consist of 3 females (A) and 3 males (B). (C‐D) Food intake (C) and AUC thereof (D) of the total mixed gender cohort (n = 3 females + 3 males). (E‐F) Survival data of Figure 1C separated by gender. (G‐I) Onset of neurological abnormalities tremors, severe tremors, and imbalance with age under AL and DR conditions when sActRIIB was administered late in life from 16 weeks of age while the DR intervention was still initiated from 8 weeks of age. ****P < 0.0001 Figure S2. Exercise profiling and muscle weights of Ercc1 Δ/− mice. (A‐B) Grip strength measure of forelimbs (A) and all limbs (B) in Newton. (C‐G) Dissected muscle weights for tibialis anterior (TA), extensor digitorum longus (EDL), gastrocnemius (Gas.), soleus (Sol.), and plantaris (Plant.), and (H‐L) normalisation of key muscle weights to body weigth. All measures from 16‐week‐old male mice. n = 4 males per group. *P < 0.05, **P < 0.01, ***P < 0.001, ****P < 0.0001. Figure S3. Organ and blood profiling of Ercc1 Δ/− mice. (A) Measure of Tibial length. (B‐E) Normalisation of Liver (B), Kidney (C), Brain (D), and Spleen (E) to body weight. (F‐J) Weights of heart (F), lung (G), stomach (H), testis (I) and thymus (J) and (K‐O) normalisation of these organs to body weight. Measure of (P) blood glucose, (Q) cholesterol, and (R) triglycerides. All measures from 16‐week‐old male mice. n = 4 males per group. *P < 0.05, **P < 0.01, ***P < 0.001, ****P < 0.0001. Figure S4. Profiling of Ercc1 Δ/− EDL muscle fibre size and MHC distribution. (A) EDL muscle fibre size profiling based on MHC expression. (B) EDL MHC composition based on MHC expression. EDL muscles from 16 week old mice. Between 55–75 fibres were counted from each mouse before bei [file JCSM-15-2361-s002.pdf]

Figure S1

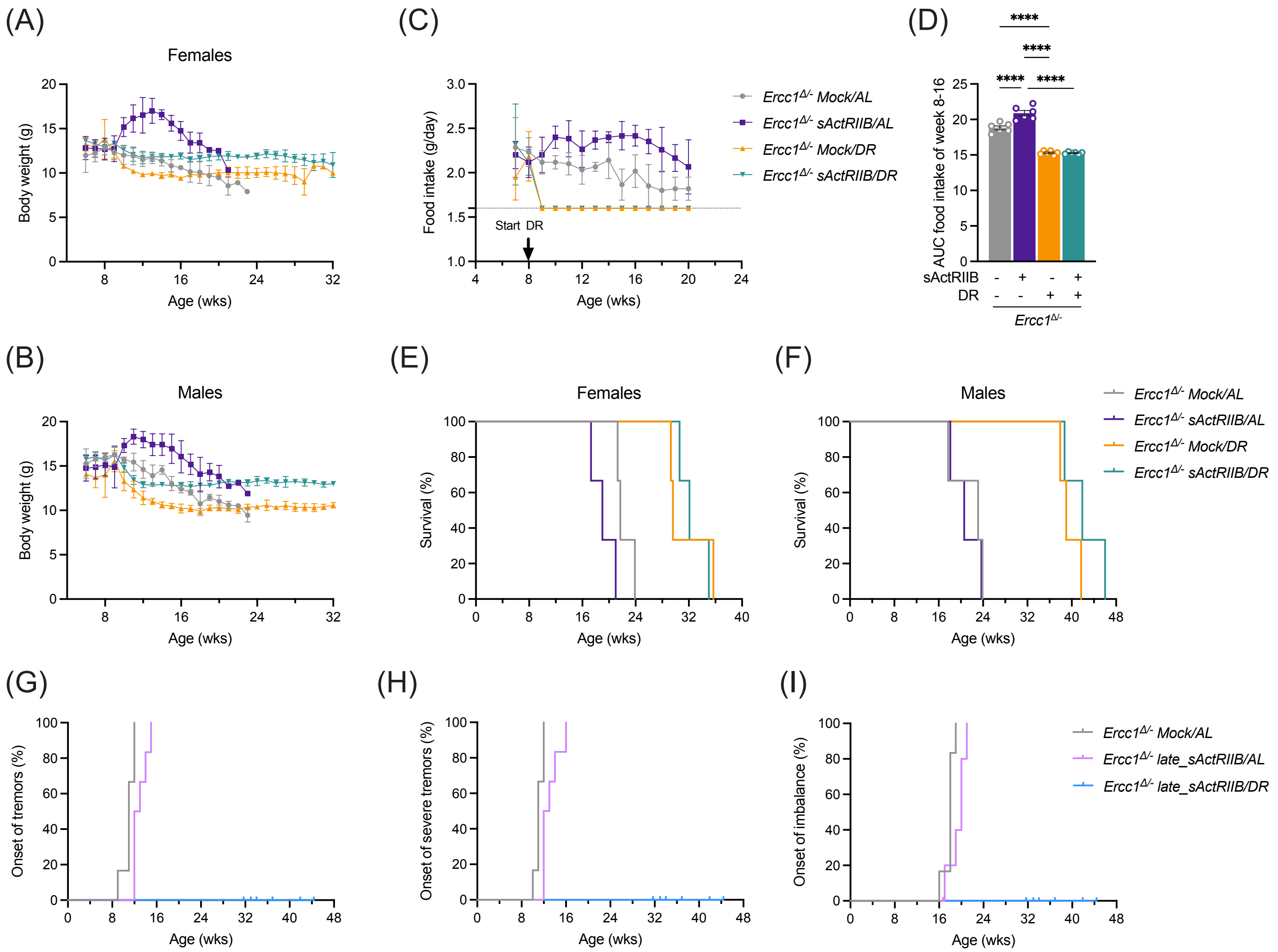
(A)
Females
Body weight (g)
Age (wks)
(B)
Males
Body weight (g)
Age (wks)
(C)
Food intake (g/day)
Start DR
Age (wks)
Ercc1Δ/- Mock/AL
Ercc1Δ/- sActRIIB/AL
Ercc1Δ/- Mock/DR
Ercc1Δ/- sActRIIB/DR
(D)
AUC food intake of week 8-16
****
sActRIIB
DR
Ercc1Δ/-
(E)
Females
Survival (%)
Age (wks)
(F)
Males
Survival (%)
Age (wks)
Ercc1Δ/- Mock/AL
Ercc1Δ/- sActRIIB/AL
Ercc1Δ/- Mock/DR
Ercc1Δ/- sActRIIB/DR
(G)
Onset of tremors (%)
Age (wks)
(H)
Onset of severe tremors (%)
Age (wks)
(I)
Onset of imbalance (%)
Age (wks)
Ercc1Δ/- Mock/AL
Ercc1Δ/- late_sActRIIB/AL
Ercc1Δ/- late_sActRIIB/DR

Figure S2

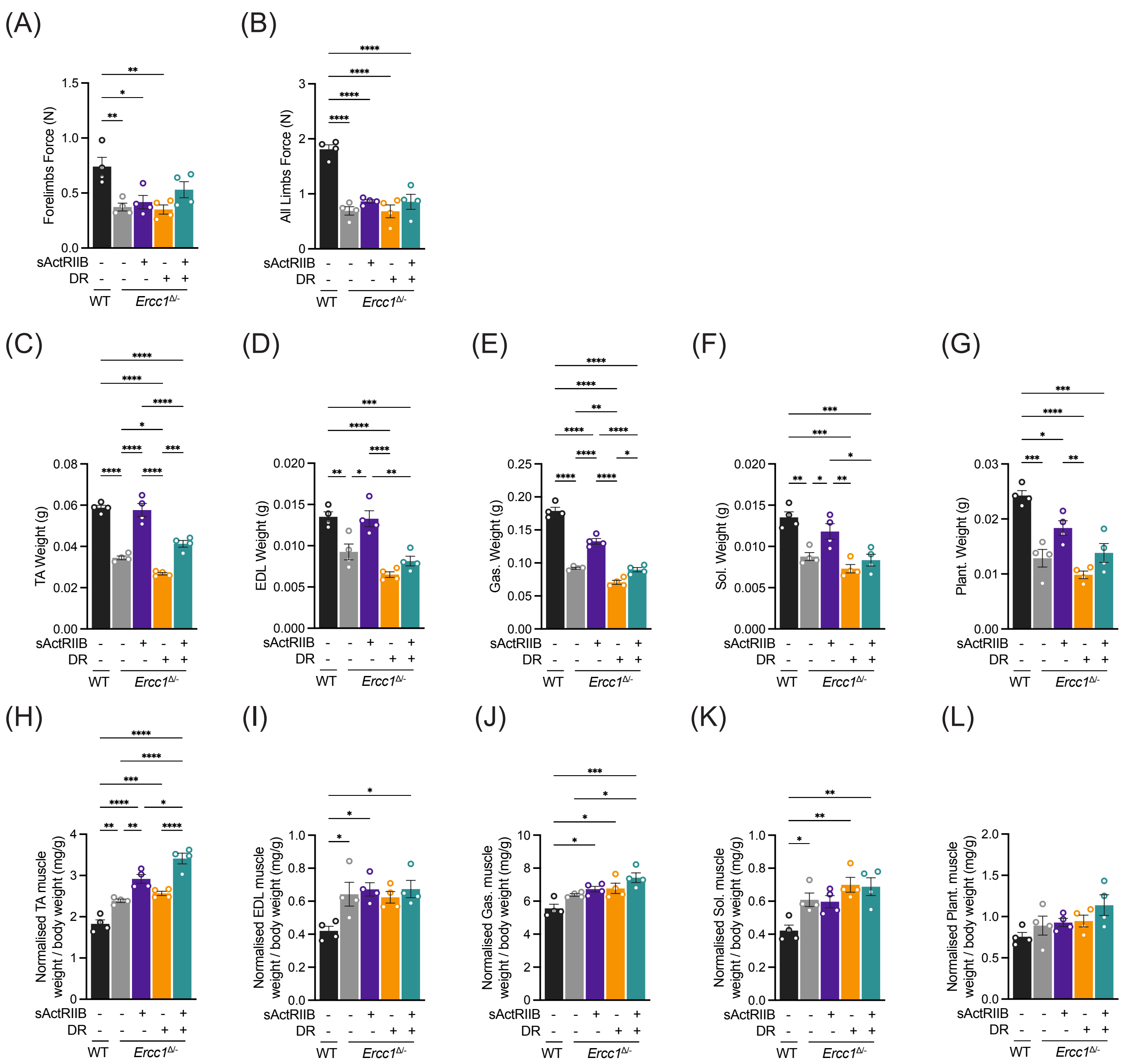

(A)
(B)
(C)
(D)
(E)
(F)
(G)
(H)
(I)
(J)
(K)
(L)
Forelimbs Force (N)
All Limbs Force (N)
TA Weight (g)
EDL Weight (g)
Gas. Weight (g)
Sol. Weight (g)
Plant. Weight (g)
Normalised TA muscle weight / body weight (mg/g)
Normalised EDL muscle weight / body weight (mg/g)
Normalised Gas. muscle weight / body weight (mg/g)
Normalised Sol. muscle weight / body weight (mg/g)
Normalised Plant. muscle weight / body weight (mg/g)
sActRIIB
DR
WT
Ercc1Δ/-

Figure S3

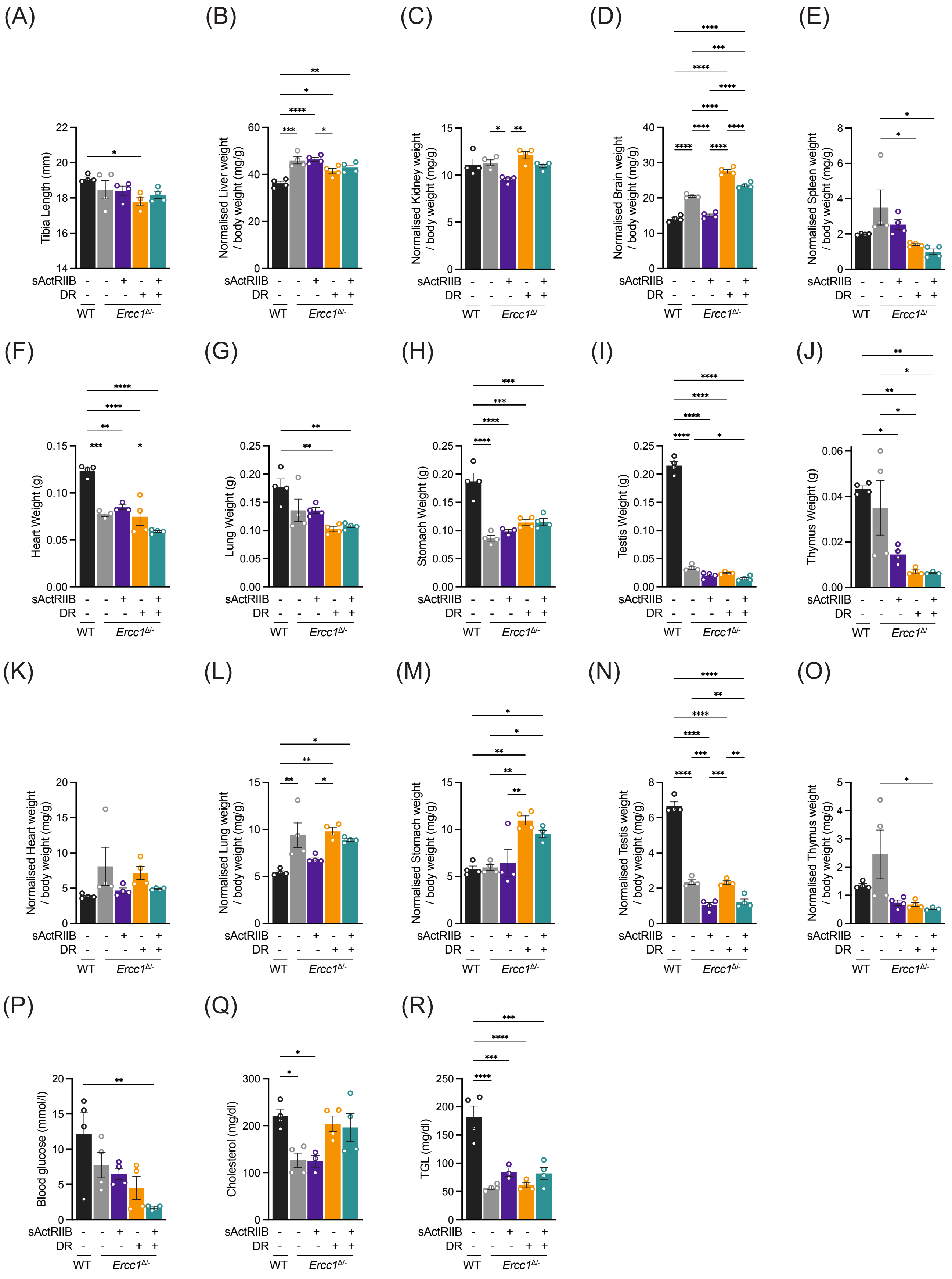

(A)
Tibia Length (mm)
(B)
Normalised Liver weight / body weight (mg/g)
(C)
Normalised Kidney weight / body weight (mg/g)
(D)
Normalised Brain weight / body weight (mg/g)
(E)
Normalised Spleen weight / body weight (mg/g)
(F)
Heart Weight (g)
(G)
Lung Weight (g)
(H)
Stomach Weight (g)
(I)
Testis Weight (g)
(J)
Thymus Weight (g)
(K)
Normalised Heart weight / body weight (mg/g)
(L)
Normalised Lung weight / body weight (mg/g)
(M)
Normalised Stomach weight / body weight (mg/g)
(N)
Normalised Testis weight / body weight (mg/g)
(O)
Normalised Thymus weight / body weight (mg/g)
(P)
Blood glucose (mmol/l)
(Q)
Cholesterol (mg/dl)
(R)
TGL (mg/dl)
sActRIIB - - + - +
DR - - - + +
WT
Ercc1Δ/-

Figure S4

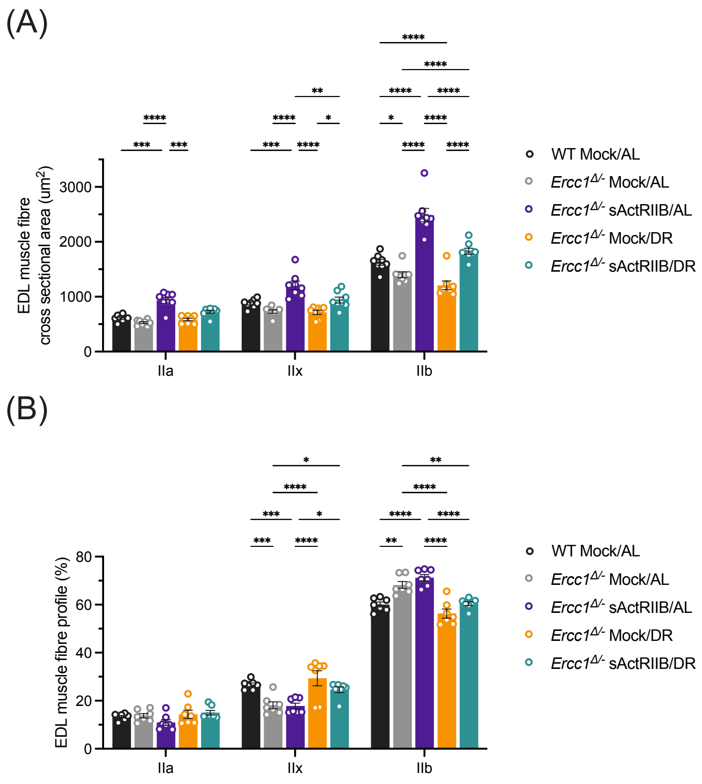

(A)
EDL muscle fibre cross sectional area (um2)
0
1000
2000
3000
IIa
IIx
IIb
WT Mock/AL
Ercc1Δ/- Mock/AL
Ercc1Δ/- sActRIIB/AL
Ercc1Δ/- Mock/DR
Ercc1Δ/- sActRIIB/DR
(B)
EDL muscle fibre profile (%)
0
20
40
60
80
IIa
IIx
IIb
WT Mock/AL
Ercc1Δ/- Mock/AL
Ercc1Δ/- sActRIIB/AL
Ercc1Δ/- Mock/DR
Ercc1Δ/- sActRIIB/DR

Figure S5

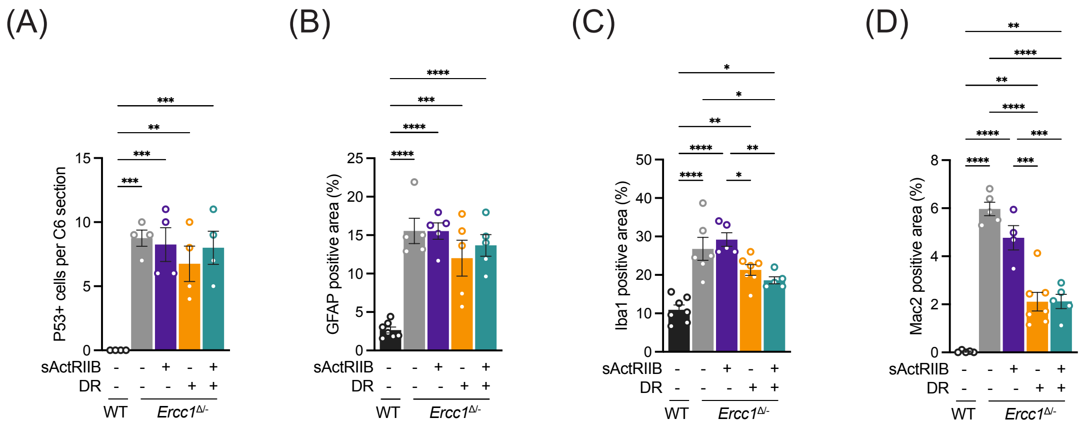
(A)
(B)
(C)
(D)
P53+ cells per C6 section
GFAP positive area (%)
Iba1 positive area (%)
Mac2 positive area (%)
sActRIIB
DR
WT
Ercc1Δ/-

Figure S6

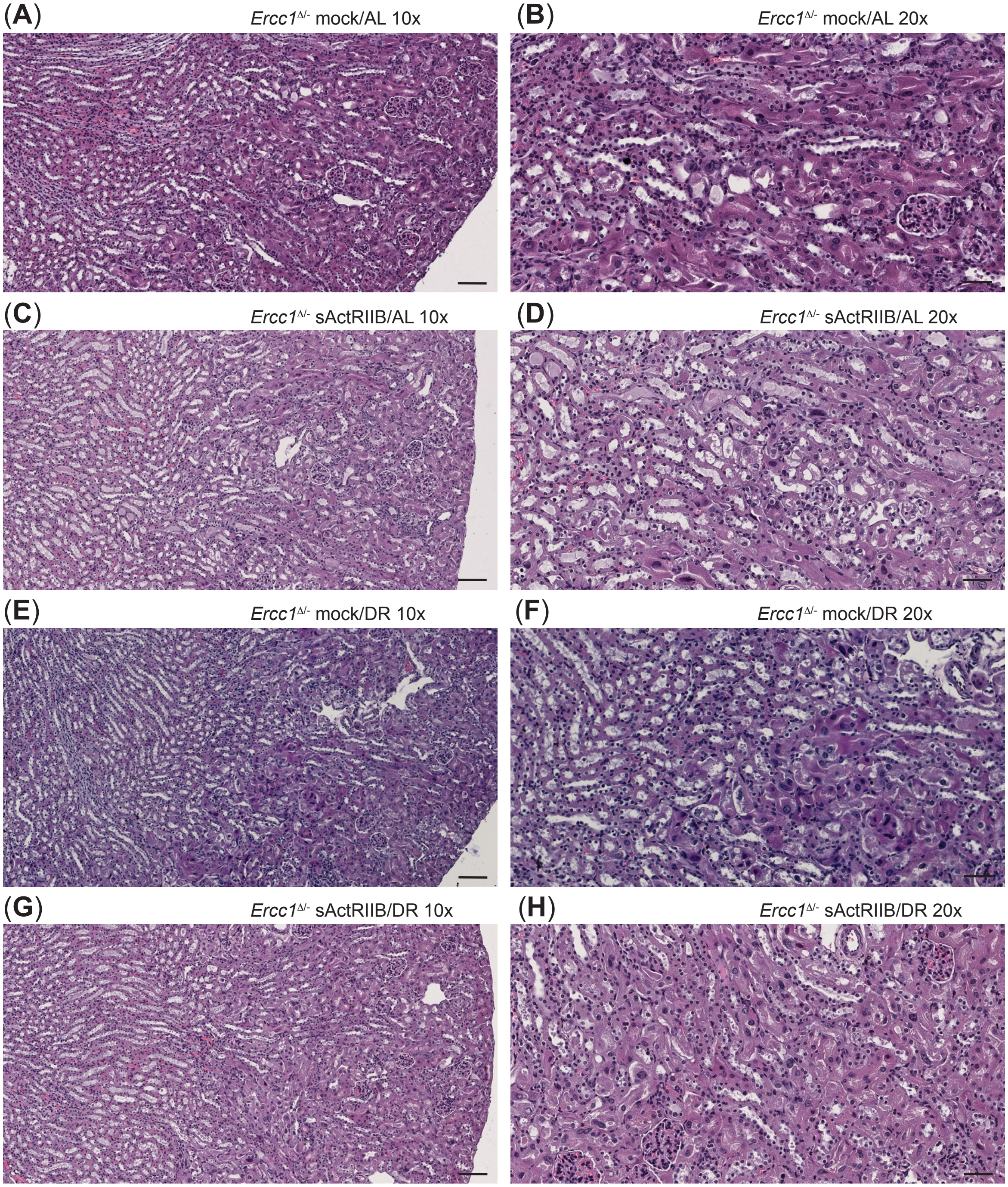
(A)
Ercc1Δ/- mock/AL 10x
(B)
Ercc1Δ/- mock/AL 20x
(C)
Ercc1Δ/- sActRIIB/AL 10x
(D)
Ercc1Δ/- sActRIIB/AL 20x
(E)
Ercc1Δ/- mock/DR 10x
(F)
Ercc1Δ/- mock/DR 20x
(G)
Ercc1Δ/- sActRIIB/DR 10x
(H)
Ercc1Δ/- sActRIIB/DR 20x

Figure S7

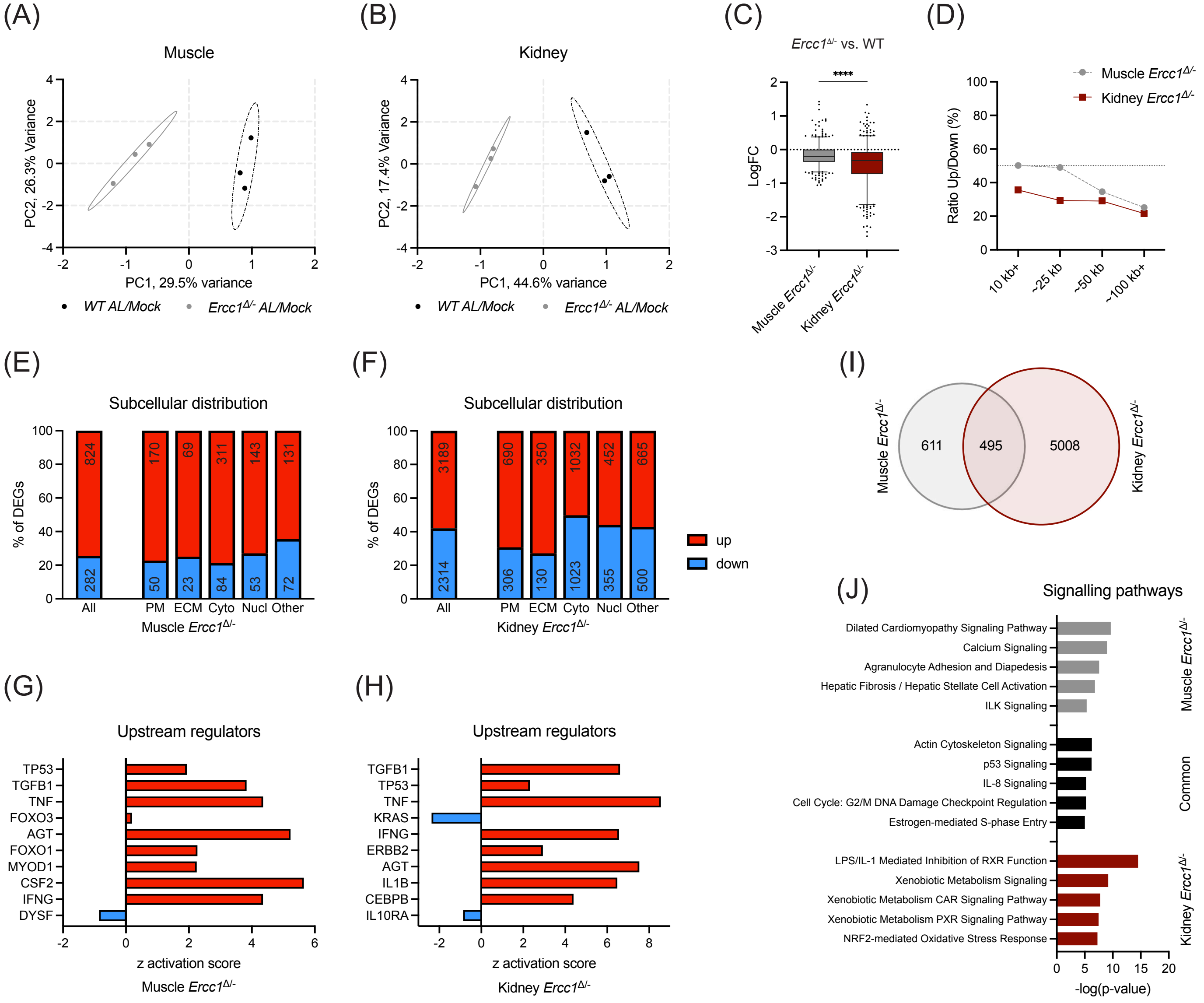
(A)
Muscle
PC2, 26.3% Variance
PC1, 29.5% variance
WT AL/Mock
Ercc1Δ/- AL/Mock
(B)
Kidney
PC2, 17.4% Variance
PC1, 44.6% variance
WT AL/Mock
Ercc1Δ/- AL/Mock
(C)
Ercc1Δ/- vs. WT
****
LogFC
Muscle Ercc1Δ/-
Kidney Ercc1Δ/-
(D)
Muscle Ercc1Δ/-
Kidney Ercc1Δ/-
Ratio Up/Down (%)
10 kb+
~25 kb
~50 kb
~100 kb+
(E)
Subcellular distribution
% of DEGs
824
170
69
311
143
131
282
50
23
84
53
72
All
PM
ECM
Cyto
Nucl
Other
Muscle Ercc1Δ/-
(F)
Subcellular distribution
% of DEGs
3189
690
350
1032
452
665
2314
306
130
1023
355
500
All
PM
ECM
Cyto
Nucl
Other
Kidney Ercc1Δ/-
up
down
(I)
Muscle Ercc1Δ/-
611
495
5008
Kidney Ercc1Δ/-
(G)
Upstream regulators
TP53
TGFB1
TNF
FOXO3
AGT
FOXO1
MYOD1
CSF2
IFNG
DYSF
z activation score
Muscle Ercc1Δ/-
(H)
Upstream regulators
TGFB1
TP53
TNF
KRAS
IFNG
ERBB2
AGT
IL1B
CEBPB
IL10RA
z activation score
Kidney Ercc1Δ/-
(J)
Signalling pathways
Dilated Cardiomyopathy Signaling Pathway
Calcium Signaling
Agranulocyte Adhesion and Diapedesis
Hepatic Fibrosis / Hepatic Stellate Cell Activation
ILK Signaling
Muscle Ercc1Δ/-
Actin Cytoskeleton Signaling
p53 Signaling
IL-8 Signaling
Cell Cycle: G2/M DNA Damage Checkpoint Regulation
Estrogen-mediated S-phase Entry
Common
LPS/IL-1 Mediated Inhibition of RXR Function
Xenobiotic Metabolism Signaling
Xenobiotic Metabolism CAR Signaling Pathway
Xenobiotic Metabolism PXR Signaling Pathway
NRF2-mediated Oxidative Stress Response
Kidney Ercc1Δ/-
-log(p-value)

Figure S8

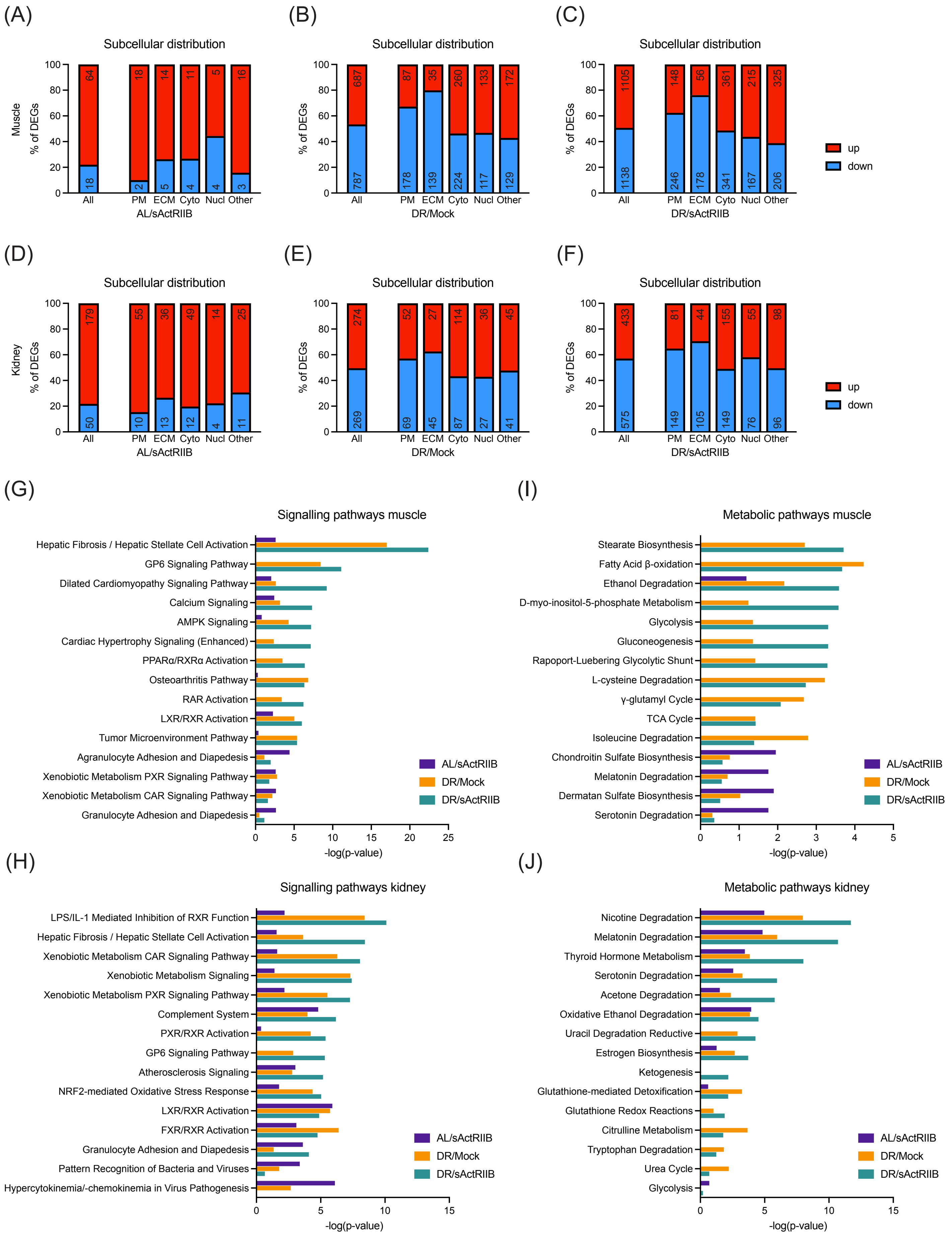
(A)
Subcellular distribution
Muscle
% of DEGs
All PM ECM Cyto Nucl Other
AL/sActRIIB
(B)
Subcellular distribution
DR/Mock
(C)
Subcellular distribution
DR/sActRIIB
up
down
(D)
Subcellular distribution
Kidney
% of DEGs
AL/sActRIIB
(E)
Subcellular distribution
DR/Mock
(F)
Subcellular distribution
DR/sActRIIB
(G)
Signalling pathways muscle
Hepatic Fibrosis / Hepatic Stellate Cell Activation
GP6 Signaling Pathway
Dilated Cardiomyopathy Signaling Pathway
Calcium Signaling
AMPK Signaling
Cardiac Hypertrophy Signaling (Enhanced)
PPARα/RXRα Activation
Osteoarthritis Pathway
RAR Activation
LXR/RXR Activation
Tumor Microenvironment Pathway
Agranulocyte Adhesion and Diapedesis
Xenobiotic Metabolism PXR Signaling Pathway
Xenobiotic Metabolism CAR Signaling Pathway
Granulocyte Adhesion and Diapedesis
-log(p-value)
AL/sActRIIB
DR/Mock
DR/sActRIIB
(I)
Metabolic pathways muscle
Stearate Biosynthesis
Fatty Acid β-oxidation
Ethanol Degradation
D-myo-inositol-5-phosphate Metabolism
Glycolysis
Gluconeogenesis
Rapoport-Luebering Glycolytic Shunt
L-cysteine Degradation
γ-glutamyl Cycle
TCA Cycle
Isoleucine Degradation
Chondroitin Sulfate Biosynthesis
Melatonin Degradation
Dermatan Sulfate Biosynthesis
Serotonin Degradation
(H)
Signalling pathways kidney
LPS/IL-1 Mediated Inhibition of RXR Function
Hepatic Fibrosis / Hepatic Stellate Cell Activation
Xenobiotic Metabolism CAR Signaling Pathway
Xenobiotic Metabolism Signaling
Xenobiotic Metabolism PXR Signaling Pathway
Complement System
PXR/RXR Activation
GP6 Signaling Pathway
Atherosclerosis Signaling
NRF2-mediated Oxidative Stress Response
LXR/RXR Activation
FXR/RXR Activation
Granulocyte Adhesion and Diapedesis
Pattern Recognition of Bacteria and Viruses
Hypercytokinemia/-chemokinemia in Virus Pathogenesis
(J)
Metabolic pathways kidney
Nicotine Degradation
Melatonin Degradation
Thyroid Hormone Metabolism
Serotonin Degradation
Acetone Degradation
Oxidative Ethanol Degradation
Uracil Degradation Reductive
Estrogen Biosynthesis
Ketogenesis
Glutathione-mediated Detoxification
Glutathione Redox Reactions
Citrulline Metabolism
Tryptophan Degradation
Urea Cycle
Glycolysis

Figure S9

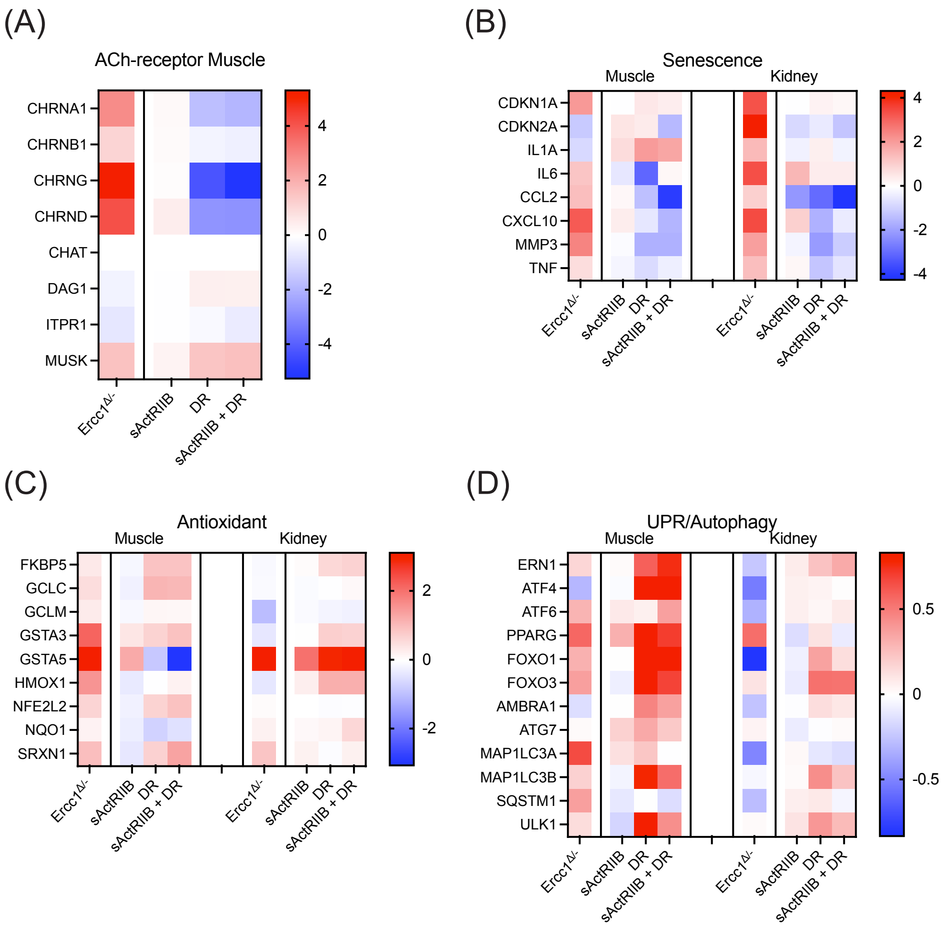

(A)
ACh-receptor Muscle
CHRNA1
CHRNB1
CHRNG
CHRND
CHAT
DAG1
ITPR1
MUSK
Ercc1Δ/-
sActRIIB
DR
sActRIIB + DR
4
2
0
-2
-4
(B)
Senescence
Muscle
Kidney
CDKN1A
CDKN2A
IL1A
IL6
CCL2
CXCL10
MMP3
TNF
Ercc1Δ/-
sActRIIB
DR
sActRIIB + DR
Ercc1Δ/-
sActRIIB
DR
sActRIIB + DR
4
2
0
-2
-4
(C)
Antioxidant
Muscle
Kidney
FKBP5
GCLC
GCLM
GSTA3
GSTA5
HMOX1
NFE2L2
NQO1
SRXN1
Ercc1Δ/-
sActRIIB
DR
sActRIIB + DR
Ercc1Δ/-
sActRIIB
DR
sActRIIB + DR
2
0
-2
(D)
UPR/Autophagy
Muscle
Kidney
ERN1
ATF4
ATF6
PPARG
FOXO1
FOXO3
AMBRA1
ATG7
MAP1LC3A
MAP1LC3B
SQSTM1
ULK1
Ercc1Δ/-
sActRIIB
DR
sActRIIB + DR
Ercc1Δ/-
sActRIIB
DR
sActRIIB + DR
0.5
0
-0.5
